# Modeling uniquely human gene regulatory function via targeted humanization of the mouse genome

**DOI:** 10.1038/s41467-021-27899-w

**Published:** 2022-01-13

**Authors:** Emily V. Dutrow, Deena Emera, Kristina Yim, Severin Uebbing, Acadia A. Kocher, Martina Krenzer, Timothy Nottoli, Daniel B. Burkhardt, Smita Krishnaswamy, Angeliki Louvi, James P. Noonan

**Affiliations:** 1 Department of Genetics, Yale School of Medicine, New Haven, CT 06510 USA; 2 Department of Comparative Medicine, Yale School of Medicine, New Haven, CT 06510 USA; 3 Yale Genome Editing Center, Yale School of Medicine, New Haven, CT 06510 USA; 4 Department of Computer Science, Yale University, New Haven, CT 06520 USA; 5 Department of Neurosurgery, Yale School of Medicine, New Haven, CT 06510 USA; 6 Department of Neuroscience, Yale School of Medicine, New Haven, CT 06510 USA; 7 Department of Ecology and Evolutionary Biology, Yale University, New Haven, CT 06520 USA; 8 Present Address: Cancer Genetics and Comparative Genomics Branch, National Human Genome Research Institute, National Institutes of Health, Bethesda, MD 20892 USA; 9 Present Address: Center for Reproductive Longevity and Equality, Buck Institute for Research on Aging, Novato, CA 94945 USA; 10 Present Address: Neuroscience Research Training Program, Department of Psychiatry, Yale School of Medicine, New Haven, CT 06510 USA; 11 Present Address: Cellarity, Cambridge, MA 02139 USA

**Keywords:** Evolutionary genetics, Evolutionary biology, RNA sequencing, Gene regulation

## Abstract

The evolution of uniquely human traits likely entailed changes in developmental gene regulation. Human Accelerated Regions (HARs), which include transcriptional enhancers harboring a significant excess of human-specific sequence changes, are leading candidates for driving gene regulatory modifications in human development. However, insight into whether HARs alter the level, distribution, and timing of endogenous gene expression remains limited. We examined the role of the HAR *HACNS1* (HAR2) in human evolution by interrogating its molecular functions in a genetically humanized mouse model. We find that *HACNS1* maintains its human-specific enhancer activity in the mouse embryo and modifies expression of *Gbx2*, which encodes a transcription factor, during limb development. Using single-cell RNA-sequencing, we demonstrate that *Gbx2* is upregulated in the limb chondrogenic mesenchyme of *HACNS1* homozygous embryos, supporting that *HACNS1* alters gene expression in cell types involved in skeletal patterning. Our findings illustrate that humanized mouse models provide mechanistic insight into how HARs modified gene expression in human evolution.

## Abstract (web summary)

Human Accelerated Regions (HARs) are prime candidates for driving the evolution of uniquely human traits. Using humanized mice, the authors show how one HAR alters gene expression during embryonic development, yielding insight into HAR function.

## Introduction

The evolution of uniquely human physical traits required human-specific genetic changes that altered development^1,2^. Discovering the locations of these changes in the genome and determining their biological impact is a major challenge. However, over the last decade comparative studies have begun to reveal potential genetic drivers underlying novel human biological features. These efforts have identified a prominent class of elements in the genome that are highly conserved across many species but show a significant excess of human-specific sequence changes^3–7^. These elements, collectively named Human Accelerated Regions (HARs), are prime candidates to encode novel human molecular functions. Many HARs act as transcriptional enhancers during embryonic development, particularly in structures showing human-specific morphological changes such as the brain and limb^7–13^. HARs have also been shown to exhibit human-specific changes in enhancer activity^7,9,10,14–19^. These findings suggest a critical contribution for HARs in human evolution and support the long-standing hypothesis that changes in developmental gene regulatory programs contribute to evolutionary innovation^20,21^.

**Fig. 1:**
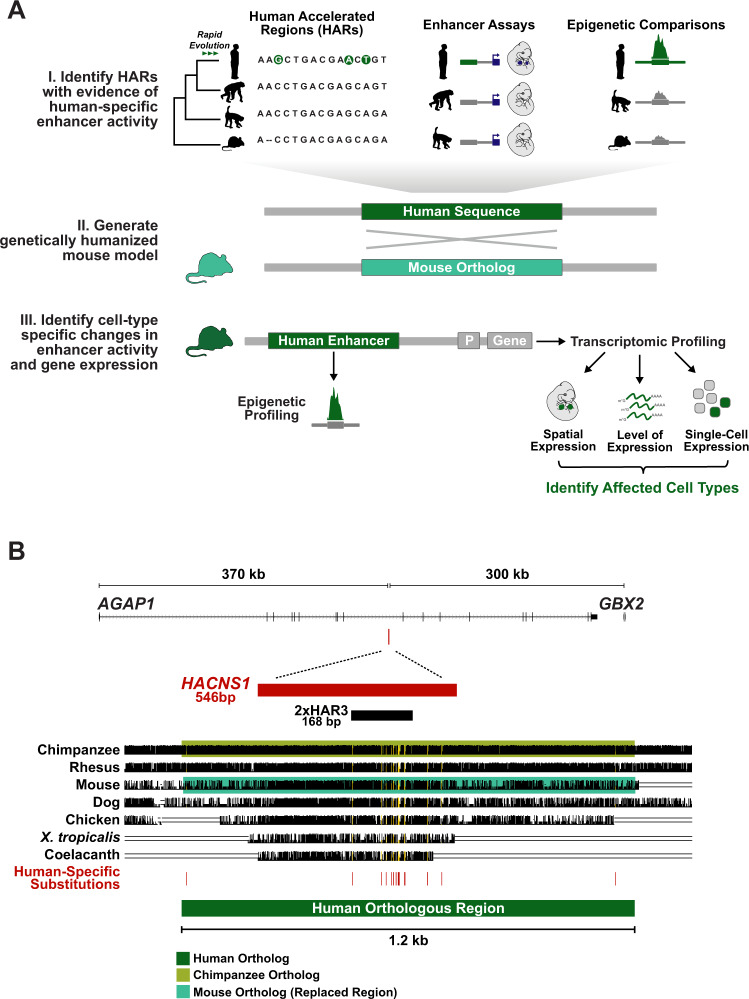
Generating a knock-in mouse model for the Human Accelerated Region *HACNS1*. **A** Schematic illustrating the generalized workflow we developed to characterize the gene regulatory functions of HARs with prior evidence of human-specific enhancer activity, which we applied to *HACNS1* in this study. **B** The location of *HACNS1* in the human genome (GRCh37/hg19) relative to the nearby genes *AGAP1* and *GBX2*. Below, alignment of the human sequence used to generate the *HACNS1* knock-in mouse with orthologous sequences from other vertebrate genomes, obtained from the UCSC hg19 100-way Multiz alignment (see Supplementary Data 1 for coordinates). The chimpanzee orthologous sequence used to generate the chimpanzee control line is highlighted in olive, and the mouse sequence replaced in each line is highlighted in teal. The location of each human-specific substitution is indicated by a red line, and the corresponding positions in the alignment are highlighted in yellow. The locations of *HACNS1* and 2xHAR3 are shown above the alignment^3,6^.

Despite these advances, the role of HARs in altering regulatory function in vivo remains poorly understood. Here we employed a knock-in approach in mouse to characterize the effects of the HAR *HACNS1* on gene expression and regulation compared to its chimpanzee ortholog during embryonic development (Fig. 1A). A similar approach has been used to model the transcriptional and developmental effects of changes in enhancer activity in other mammalian lineages, notably bats^22^. We chose to model *HACNS1* (also known as HAR2 and 2xHAR.3), as it exhibits the strongest acceleration signature of any noncoding HAR yet identified (Fig. 1B). *HACNS1* was also the first HAR demonstrated to exhibit a human-specific gain in enhancer activity during development. In a mouse transgenic enhancer assay, *HACNS1* was shown to drive increased expression of a *LacZ* reporter gene in the embryonic mouse limb compared to its chimpanzee and rhesus macaque orthologs^10^. Furthermore, *HACNS1* exhibits increased levels of histone H3K27 acetylation (H3K27ac), which is correlated with enhancer activity, in human versus rhesus macaque and mouse embryonic limb^11^. Together, these findings suggest that *HACNS1* may have contributed to changes in limb development during human evolution.

To compare the functions of *HACNS1* and its chimpanzee and mouse orthologs in the same developmental system, we used homologous recombination to replace the endogenous mouse sequence with the human or chimpanzee counterpart. We found that *HACNS1* maintains its human-specific enhancer activity in the mouse embryo and alters the expression of the nearby transcription factor encoding gene *Gbx2* in limb chondrocytes, a cell type required for skeletal morphogenesis. Our findings support that HARs can direct changes in endogenous gene expression during development and illustrate the power of genetically humanized mouse models to provide insight into regulatory pathways and developmental mechanisms modified in human evolution.

## Results

### Generating a *HACNS1* knock-in mouse model

We designed a targeting construct for homologous recombination including a 1.2 kb human sequence encompassing *HACNS1* that was previously shown to encode human-specific enhancer activity in transgenic mouse embryos^10^. We replaced the orthologous mouse locus using homology-directed repair in C57BL6/J-*A*^*w−J*^/J (B6 agouti) embryonic stem (ES) cells (Fig. 1B, Supplementary Figs. 1A, B, Supplementary Data 1; Methods). To provide a control that would enable us to distinguish bona fide human-specific functions of *HACNS1* from possible primate-rodent differences, we also generated a mouse model for the orthologous chimpanzee sequence using the same approach (Supplementary Figs. 1A–C). The 1.2 kb chimpanzee sequence shows no evidence of evolutionary acceleration and includes 22 single nucleotide differences relative to the human sequence^3^; 15 of these are human-specific based on comparisons to other primate genomes (Methods, Supplementary Data 2). Previous studies indicate that multiple human-specific substitutions contribute to the gain of function in *HACNS1*^10^. Twelve of the 15 substitutions introduce one or more predicted transcription factor binding sites to the human sequence (Supplementary Data 2, Supplementary Fig. 1A). An extensive comparison of sequence conservation and divergence among the human, chimpanzee, and mouse sequences is provided in the Supplementary Note (Supplementary Materials).

In order to verify the integrity of the edited loci, we sequenced a 40 kb region encompassing the human or chimpanzee sequence replacement, the homology arms used for targeting, and flanking genomic regions in mice homozygous for either *HACNS1* or the chimpanzee ortholog (Supplementary Fig. 1C; Methods). We found no evidence of aberrant editing, sequence rearrangements, or other off-target mutations at either edited locus. We also verified that each homozygous line carried two copies of the human or chimpanzee sequence using quantitative real-time PCR (RT-qPCR) (Supplementary Fig. 1D; associated Source Data).

### *HACNS1* maintains its human-specific enhancer activity in the mouse embryo

**Fig. 2:**
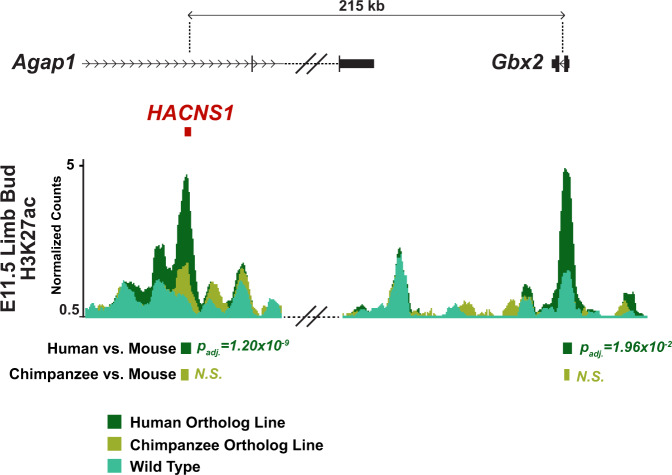
Epigenetic signatures of increased activity at *HACNS1* and the *Gbx2* promoter in the *HACNS1* homozygous mouse limb bud. Epigenetic profiling in the *HACNS1* homozygous E11.5 limb bud compared to the chimpanzee ortholog line and wild type. The normalized H3K27ac signals are shown for the *HACNS1* line (in dark green), the chimpanzee ortholog line (in olive), and wild type (in teal) (see “Methods”). The location of the edited *HACNS1* locus in the human ortholog line relative to nearby genes is shown above the track. The double slanted lines indicate intervening H3K27ac signal data between the edited and wild type loci and *Gbx2* that were removed for clarity; see Supplementary Fig. 2 for complete views for each line as well as input signals. H3K27ac peak calls showing significant increases in signal between *HACNS1* homozygous and wild type, and the corresponding peak regions compared between the chimpanzee control line and wild type, are shown below the signal track. Litter-matched embryos were used for each comparison (see “Methods”). N.S. not significant. All peak calls for each line are shown in Supplementary Fig. 2. Adjusted *P* values were obtained using DESeq2 (implemented in HOMER) with a Wald test followed by Benjamini-Hochberg correction^23,24^.

We used chromatin immunoprecipitation (ChIP) to determine if *HACNS1* exhibits epigenetic signatures of increased enhancer activity in mice. We first performed epigenetic profiling in the developing mouse limb bud based on prior evidence that *HACNS1* drives increased reporter gene activity in transgenic enhancer assays and exhibits increased H3K27ac marking in the human embryonic limb^10,11^. We profiled both H3K27ac and H3K4 dimethylation (H3K4me2), which is also associated with enhancer activity, in embryonic day (E) 11.5 limb buds from embryos homozygous for *HACNS1*, embryos homozygous for the chimpanzee ortholog, and wild type embryos. We found a strong signature of H3K27ac marking at *HACNS1* in the limb buds of *HACNS1* homozygous embryos (Fig. 2, Supplementary Fig. 2). The chimpanzee and mouse sequences both showed significant but weaker H3K27ac enrichment relative to the human sequence, supporting the conclusion that *HACNS1* maintains its human-specific enhancer activity in the mouse genomic and developmental context.

**Fig. 3:**
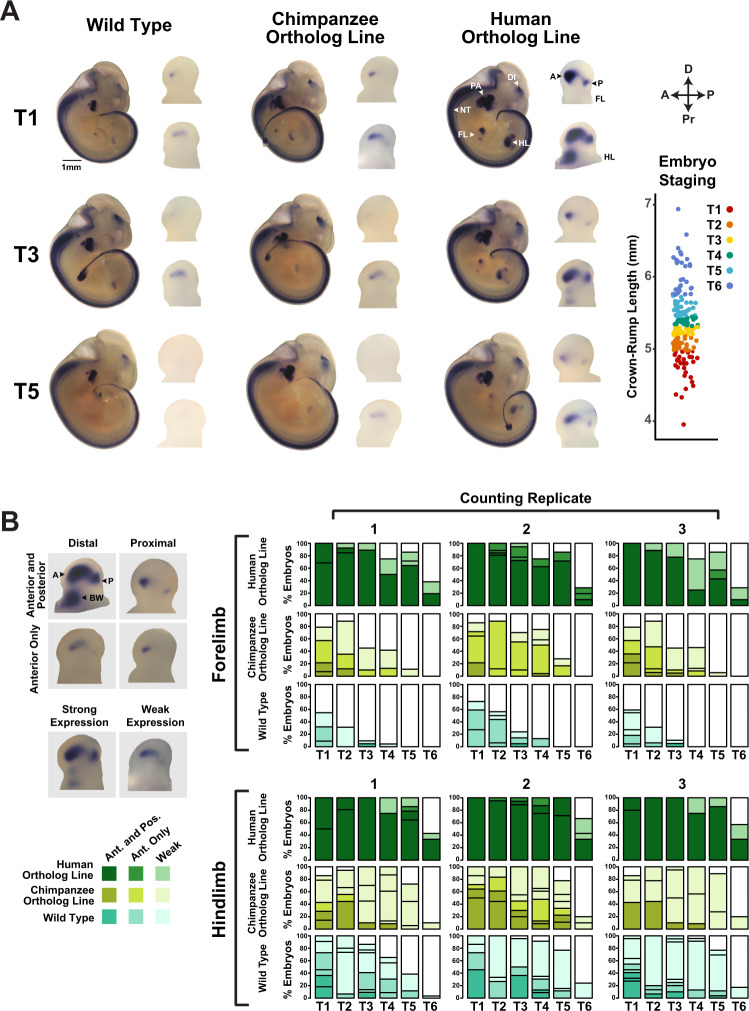
Spatial and temporal changes in *Gbx2* expression driven by *HACNS1* in *HACNS1* knock-in mouse embryos. **A** Spatial and temporal expression of *Gbx2* in *HACNS1* homozygous, chimpanzee ortholog line, and wild type E11-E12 embryos visualized by whole-mount in situ hybridization (ISH). Representative images are shown for each genotype at three fine-scale time points; see text and associated Source Data for details on staging. Magnified views of *Gbx2* expression in limb buds are shown to the right of each embryo. Annotations of anatomical structures and developmental axes are indicated at the top right: FL forelimb, HL hindlimb, DI diencephalon, NT neural tube, PA pharyngeal arch, A anterior, P posterior. The arrows at the top far right indicate the anterior-posterior (A-P) and proximal-distal (Pr-D) axes for the magnified limb buds. Bottom right: Crown-rump lengths for all embryos assayed for *Gbx2* mRNA expression by ISH. Each point indicates a single embryo. Colors denote each fine-scale time point (T1-T6). **B** *Left:* representative images of anterior, posterior, proximal, distal (top), and strong versus weak *Gbx2* staining patterns (bottom). Anterior (A), posterior (P), and body wall (BW) domains are denoted on top left limb bud. *Right: Gbx2* ISH staining pattern data across 6 developmental timepoints from each of three independent, blinded scorers (marked at top as counting replicates 1–3; see text and Fig. 3A for timepoint scheme and associated Source Data for annotations). The darkest shade for *HACNS1* homozygous (dark green), chimpanzee ortholog line (olive), and wild type (teal) represents percentage of forelimbs or hindlimbs showing strong anterior and posterior limb bud staining. Medium-dark shade, as shown in the legend on the left, denotes strong anterior staining only, while the lightest shade denotes weak staining in any domain. Independent biological specimens were analyzed for *n* = 139 (wild type), *n* = 103 (chimpanzee ortholog line), and *n* = 106 (human ortholog line) forelimbs and *n* = 137 (wild type), *n* = 103 (chimpanzee ortholog line), and *n* = 102 (human ortholog line) hindlimbs. For body wall and pharyngeal arch scoring data see Supplementary Fig. 3A, B and associated Source Data.

We used DESeq2 implemented in HOMER (Methods) to identify genome-wide significant differences in H3K27ac and H3K4me2 levels in E11.5 limb buds from mice homozygous for *HACNS1* or the chimpanzee ortholog versus wild type^23,24^. We found that H3K27ac and H3K4me2 levels were significantly increased at the edited *HACNS1* locus compared to the endogenous mouse locus (Fig. 2, Supplementary Fig. 2A, B, Supplementary Data 3). In contrast, the level of H3K27ac at the edited chimpanzee locus was not significantly different than that at the endogenous locus (Fig. 2, Supplementary Fig. 2A, Supplementary Data 3). The levels of H3K4me2 were significantly increased at both the humanized and orthologous chimpanzee loci in each respective line compared to mouse (Fig. 2, Supplementary Fig. 2, Supplementary Data 3). As high levels of H3K4me2 coupled with low levels of H3K27ac are associated with weak enhancer activity^25^, it is likely that the chimpanzee sequence is not acting as a strong enhancer in the limb bud overall, a finding further supported by the gene expression analyses described in Fig. 3.

Previous transgenic mouse enhancer assays indicated that *HACNS1* drives increased reporter gene activity in the pharyngeal arch compared to the chimpanzee ortholog^10^. We therefore profiled H3K27ac and H3K4me2 in pharyngeal arch tissue from E11.5 embryos homozygous for either *HACNS1* or the chimpanzee ortholog. We detected reproducible, significant enrichment of H3K27ac in the pharyngeal arch at the humanized and orthologous chimpanzee loci compared to input controls, but the H3K27ac signal at neither the human nor the chimpanzee ortholog locus was significantly different compared to the mouse endogenous locus (Supplementary Fig. 2). We did, however, identify a significant gain of H3K4me2 signal in the pharyngeal arch at the humanized and orthologous chimpanzee loci compared to the mouse locus (Supplementary Fig. 2, Supplementary Data 3).

In order to identify downstream epigenetic changes resulting from *HACNS1* activation in the limb bud, we searched for other genome-wide gains of H3K27ac and H3K4me2 at enhancers and promoters. We identified a significant gain of H3K27ac in *HACNS1* versus wild type limb buds at the promoter of the nearby gene *Gbx2* (Fig. 2, Supplementary Fig. 2, Supplementary Data 3). While significant H3K27ac enrichment was found in all three lines at the *Gbx2* promoter compared to input controls, H3K27ac levels were not significantly increased at *Gbx2* in limb buds with the chimpanzee ortholog compared to wild type, indicating the gain of activity is specific to *HACNS1*. H3K4me2 was also enriched at the promoter of *Gbx2* in all three lines compared to input controls (Supplementary Fig. 2B). After multiple testing correction, we did not identify any significant differentially marked regions outside of the *HACNS1-Gbx2* locus between either the *HACNS1* homozygous or the chimpanzee ortholog line compared to wild type for each chromatin mark in either tissue (Supplementary Data 3). Moreover, we did not detect a significant increase in either H3K27ac or H3K4me2 levels at the *Gbx2* promoter in the pharyngeal arch in *HACNS1* homozygous embryos (Supplementary Figs. 2C, D). This may be due to a lack of statistical power to detect small differences in histone modification levels given the number of replicates in the analysis, or our use of whole tissues to map histone modification profiles, which could obscure spatially restricted changes.

*Gbx2* encodes a transcription factor with a highly complex expression pattern and multiple functions in the developing embryo. GBX2 has been implicated in midbrain and hindbrain development^26,27^, guidance of thalamocortical projections^28,29^, ear development^30^, and pharyngeal arch patterning^31^. *Gbx2* is expressed in developing mouse limb at E10.5; however, its role in limb development remains undetermined as no limb phenotype has been reported in *Gbx2* knockout mice^27^. *HACNS1* and *GBX2* are located in the same topologically associated domain (TAD), and TADs have been shown to restrict enhancer interactions to genes within their boundaries^32,33^. In E11.5 limbs from *HACNS1* homozygous mice, the only significant increases in H3K27ac we detected in this TAD were at the *HACNS1* knock-in locus and at the endogenous *Gbx2* promoter (Supplementary Data 3). Together, these results suggest that *Gbx2* is a regulatory target of *HACNS1*, evoking the hypothesis that the gain of function in *HACNS1* might alter *Gbx2* expression in the limb.

### *HACNS1* drives spatial and quantitative changes in *Gbx2* expression in the limb bud

To visualize potential expression changes resulting from *HACNS1*-driven upregulation of the *Gbx2* promoter in *HACNS1* homozygous mouse embryos, we used in situ hybridization (ISH) (Fig. 3). We analyzed *Gbx2* expression in >50 E11.5 embryos per genotype (Fig. 3A, B; Methods and associated Source Data). In wild type embryos, we observed single foci of *Gbx2* expression in forelimb and hindlimb (Fig. 3A, left). In contrast, embryos homozygous for *HACNS1* showed substantially increased *Gbx2* expression in both forelimb and hindlimb (Fig. 3A, right). Embryos homozygous for the chimpanzee ortholog showed a weak increase in *Gbx2* expression compared to wild type (Fig. 3A, B). *Gbx2* expression in *HACNS1* homozygous embryos was increased in two distinct anterior and posterior regions in the forelimb and hindlimb bud, as well as an anterior proximal region in the latter. Overall, *Gbx2* expression in the limb bud was temporally dynamic in embryos of all genotypes. Embryos from the same litter vary in developmental age such that individual embryos collected at E11.5 range from E11 to E12. Therefore, we established a fine staging scheme to characterize changes in *Gbx2* expression within this short developmental interval. We assigned embryos to 6 temporally ordered groups (designated T1-T6, and ranging from ~40 to 48 somites, although we did not use somite counts to stage embryos; see Methods for more details) according to crown-rump length and used a blinded approach to qualitatively assess staining patterns (Fig. 3A, B and associated Source Data)^10,34–36^.

We identified differences in the distribution of *Gbx2* expression in the forelimb and hindlimb buds of *HACNS1* compared to both chimpanzee ortholog and wild type embryos across all 6 developmental time points (Fig. 3B). At the earliest time point (T1), we found that *Gbx2* was strongly expressed in distinct anterior-distal and posterior domains in *HACNS1* forelimb and hindlimb buds (Fig. 3A, B). Robust expression of *Gbx2* in *HACNS1* limb buds persisted through the remaining time points (up to T6), though the size of the anterior and posterior domains decreased over time. Strong expression of *Gbx2 in HACNS1* homozygous embryos persisted for a longer period of time in hindlimb than in forelimb, consistent with the delayed developmental maturation of the former^37^. In addition, *HACNS1* homozygous embryos showed a hindlimb-specific anterior-proximal expression domain adjacent to the body wall across all 6 time points (Fig. 3A, B, Supplementary Fig. 3A).

In contrast to the robust expression observed in limb buds of *HACNS1* homozygous mice, *Gbx2* expression in limb buds from both the chimpanzee ortholog and wild type lines was weak and mostly evident at early time points (Fig. 3). Chimpanzee ortholog line embryos and wild type embryos both showed weak distal *Gbx2* expression foci in early forelimb and hindlimb that were generally restricted to the anterior limb bud (Fig. 3A, B). Weak distal expression was primarily restricted to approximately T1-T2 in wild type forelimb but persisted until approximately T4 in a subset of embryos with the chimpanzee ortholog (Fig. 3A, B). Weak distal expression persisted in hindlimb through T5-T6 in both the chimpanzee line and wild type (Fig. 3B, bottom). These findings suggest that the chimpanzee ortholog line exhibits a modest increase in *Gbx2* expression compared to wild type, potentially due to primate-rodent sequence differences affecting enhancer activity that our experimental design was intended to control for (see Supplemental Note). However, the *HACNS1* knock-in line exhibits profound changes in *Gbx2* limb bud expression compared to both. Together, these findings suggest that *HACNS1* drives spatial and quantitative changes in *Gbx2* expression in the limb, as well as a temporal extension of expression compared to wild type.

In addition to the forelimb and hindlimb bud, *Gbx2* was also expressed in the neural tube, diencephalon, and pharyngeal arches of embryos homozygous for *HACNS1* or the chimpanzee ortholog, and in wild type embryos (Fig. 3A)^27,29,31^. Whereas *Gbx2* expression was primarily restricted to the first pharyngeal arch in embryos with the chimpanzee ortholog and in wild type embryos, it expanded dorsally into the second pharyngeal arch in *HACNS1* homozygous embryos during T1-T5 (Supplementary Fig. 3B). However, we chose to focus on limb, due to the absence of a significant gain in H3K27ac marking at the *HACNS1* knock-in locus or the *Gbx2* promoter in *HACNS1* homozygous versus wild type pharyngeal arch (Supplementary Fig. 2).

In order to quantify the gain of *Gbx2* expression in *HACNS1* knock-in mice, we used real-time quantitative reverse transcription PCR (RT-qPCR) in pooled forelimb and hindlimb buds from embryos homozygous for *HACNS1*, the chimpanzee ortholog, and the endogenous mouse locus at time points T1-T6. We found that *Gbx2* expression was increased in forelimb and hindlimb of *HACNS1* embryos versus both chimpanzee ortholog and wild type at all 6 time points (Supplementary Fig. 3C and associated Source Data). Although we detected an increase in *Gbx2* expression in forelimb and hindlimb of embryos with the chimpanzee ortholog versus wild type at early time points, this change was substantially weaker than that between *HACNS1* and wild type or *HACNS1* and the chimpanzee line. Consistent with our ISH results, *Gbx2* expression in *HACNS1* knock-in line forelimb and hindlimb was strongest at the earliest time points and persisted longer in hindlimb than in forelimb (Fig. S3C). While *Gbx2* expression declined over time in all three genotypes, it persisted longer in *HACNS1* knock-in line forelimb and hindlimb.

### *HACNS1* drives increased *Gbx2* expression in limb chondrogenic mesenchymal cells

In order to identify the specific cell types expressing *Gbx2* as well as genes that are co- expressed with *Gbx2* in the developing limb of all three genotypes, we performed single-cell RNA-sequencing (scRNA-seq) in E11.5 hindlimbs, which showed the most pronounced upregulation of *Gbx2* in spatial and quantitative expression analyses (Fig. 3, Supplementary Fig. 3). Using the 10x Genomics scRNA-seq platform for cell barcoding, library preparation, and sequencing, we obtained transcriptomes from ~10,000 cells per genotype. We used the Seurat toolkit for data preprocessing and library size normalization (Methods)^38^. During pre-processing, we removed endothelial and blood cells (*Cd34*-positive; *Pf4*-positive; *Hbb*-positive), as our analysis indicated that these developmentally and transcriptionally distinct cell types do not express *Gbx2* in any of our datasets^39,40^. After normalization of the filtered data using the SCTransform method in Seurat and integration of data from all samples into a single dataset using the Seurat v3 integration workflow (Methods), we performed clustering analysis on the integrated dataset to identify cell type categories present in all three genotypes^38^. To visualize similarities between cells, we used Uniform Manifold Approximation and Projection (UMAP), a dimensionality reduction method for data visualization, followed by the Louvain method for community detection to identify cell subtypes^41,42^.

**Fig. 4:**
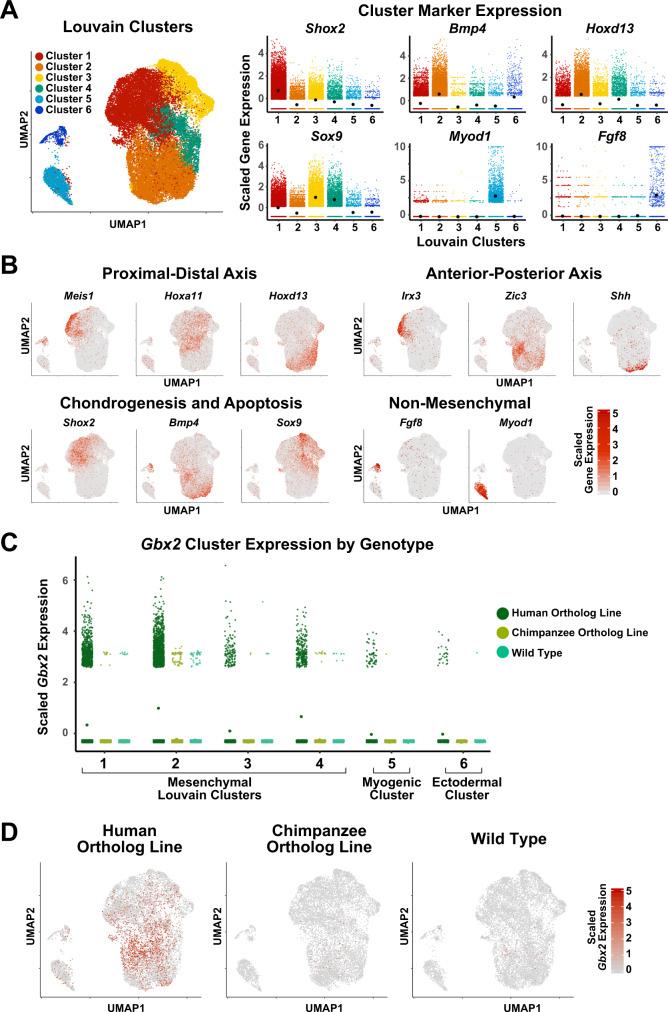
Single-cell transcriptome analysis of E11.5 hindlimb bud in *HACNS1* knock-in, chimpanzee ortholog line, and wild type embryos. **A** Left: UMAP embedding of *HACNS1* homozygous, chimpanzee ortholog line, and wild type cells. The colors indicate cell clusters identified by Louvain clustering. Right: Expression of known limb bud cell-type marker genes in each cluster. Black dots denote cluster mean expression. **B** UMAP embedding of hindlimb bud cells from *HACNS1* homozygous, chimpanzee ortholog line, and wild type, showing expression of proximal-distal, anterior-posterior, chondrogenesis-apoptosis, and non-mesenchymal markers. See text and Supplementary Fig. 4 for details. **C** Expression of *Gbx2* in each Louvain cluster, separated by genotype. Dots denote cluster mean expression. **D** UMAP embeddings illustrating cells expressing *Gbx2* (indicated in red) in *HACNS1* homozygous, chimpanzee ortholog line, and wild type cells. All gene expression data shown in plots and UMAP embeddings (**A**–**D**) were imputed using ALRA and centered and scaled using z-scores (see “Methods”)^81^.

This analysis revealed three distinct groups: (a) mesenchymal cell subtypes based on expression of the known markers *Sox9* (clusters 1, 3, 4), *Bmp4* (cluster 2)*, Shox2* (cluster 1), and *Hoxd13* (clusters 1–4) (Fig. 4A); (b) non-mesenchymal cell types, including myogenic cells (cluster 5, *Myod*); and (c) ectodermal cells (cluster 6, *Fgf8*) (Fig. 4A)^43–48^. Furthermore, our analysis revealed finer separation of mesenchymal cells according to known limb patterning markers. We first examined the expression of known proximal-distal limb bud markers *Meis1, Hoxa11*, and *Hoxd13* (proximal, medial, and distal, respectively)^49^. Cells expressing each of these markers showed a distinct localization in the UMAP embedding (*Meis1* + cells in the top left, *Hoxa11* + cell in the center, and *Hoxd13* + cells in the lower right; Fig. 4B, upper left), suggesting our analysis recovered transcriptional and cell-type transitions along the proximal-distal patterning axis (Fig. 4B).

We also found that the first axis of the UMAP embedding clearly recapitulated known gene expression gradients along the anterior-posterior limb bud axis based on expression of the anterior-proximal marker *Irx3*, the anterior marker *Zic3*, and the posterior-proximal marker *Shh* (Fig. 4B, upper right)^43,50–52^. Using markers of chondrogenic (*Sox9, Shox2*) versus non-chondrogenic (*Bmp4*) mesenchyme, we found that the second UMAP axis followed the chondrogenic versus interdigital apoptotic fate gradient (Fig. 4B)^43,45,48^. We also found that the expression patterns of these markers were broadly conserved between genotypes, with each genotype showing comparable subsets of proximal, distal, anterior, posterior, chondrogenic, and non-chondrogenic cell types (Supplementary Figs. 4A, B). Collectively, our scRNA-seq analyses identified specific conserved cell types and spatial transcriptional gradients in the developing hindlimb bud across all three genotypes.

We then sought to define genotype-specific differences in *Gbx2* expression. In order to identify the cell types expressing *Gbx2* in *HACNS1* homozygous hindlimb buds, we examined the distribution of *Gbx2*-positive cells across cell clusters in all three genotypes. We found that *Gbx2* was upregulated in *HACNS1* homozygous hindlimbs versus chimpanzee ortholog and wild type hindlimbs, primarily in the mesenchymal cell clusters (clusters 1–4), consistent with the ISH and RT-qPCR expression analyses (Fig. 3, Supplementary Fig. 3C and Fig. 4C). In *HACNS1* homozygous hindlimbs, 24% of cells expressed *Gbx2*, 96% of which were mesenchymal cells, whereas less than 1% of cells in chimpanzee ortholog and wild type hindlimbs expressed *Gbx2* (Methods; Supplementary Data 4). Greater than 98% of *Gbx2*-positive cells in the chimpanzee ortholog line and wild type hindlimb were mesenchymal, and the majority of *Gbx2*-positive cells in each of these lines were assigned to Louvain cluster 2 (70% and 68%, respectively; Supplementary Data 4). Only one non-mesenchymal cell from chimpanzee ortholog hindlimb and one non-mesenchymal cell from wild type hindlimb was *Gbx2-*positive (Fig. 4C; Supplementary Data 4). UMAP embedding of cells revealed that *Gbx2-*positive cells in *HACNS1* homozygous hindlimb buds largely clustered within a distinct subset of mesenchymal cells belonging primarily to Louvain clusters 1, 2 and 4 (Fig. 4C, D; Supplementary Data 4). The genotype-specific differences in *Gbx2* expression were consistent between the imputed and unimputed data as well as across individual replicates (Supplementary Fig. 4C, D).

**Fig. 5:**
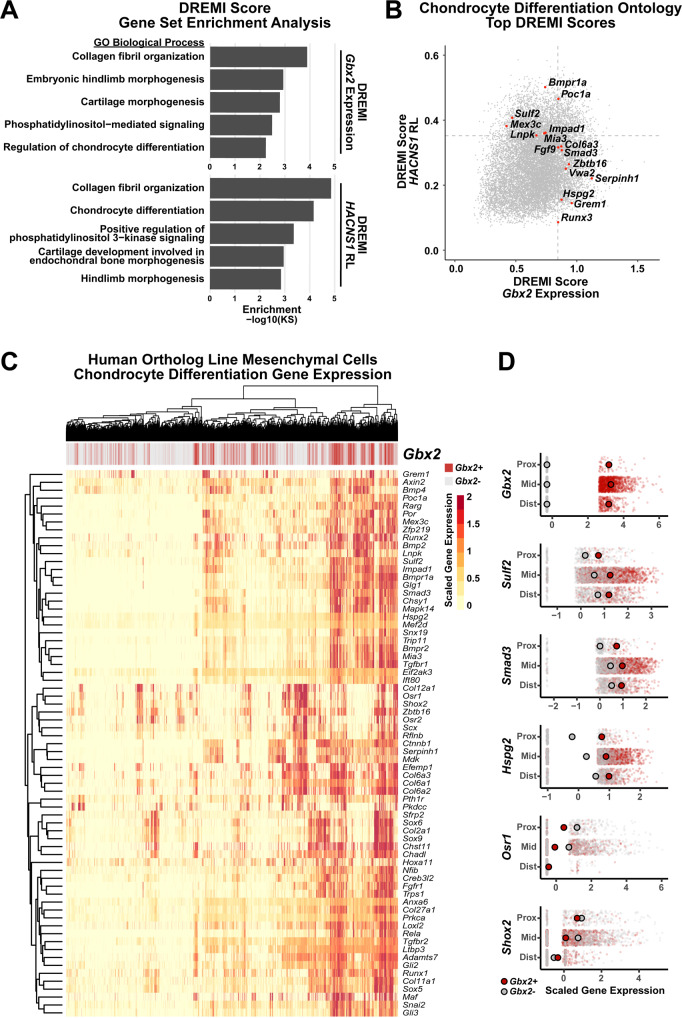
*Gbx2-*positive mesenchymal cell expression of chondrocyte differentiation markers in *HACNS1* homozygous limb bud. **A** Ontology enrichments of genes with expression associated with *Gbx2* expression (top) and the relative likelihood of the *HACNS1* knock-in condition (*HACNS1* RL, bottom) in *HACNS1* homozygous mesenchymal cells. The log-transformed Gene Set Enrichment Analysis Kolmogorov–Smirnov *P* value for each category is plotted on the x-axis. Ontologies shown are those overlapping in the *Gbx2* expression and *HACNS1* RL ontology enrichment lists. See also Supplementary Data 5 and 6. **B** *HACNS1* RL and *Gbx2* kNN-DREMI scores are plotted for all genes. Genes ranked in the top 20% of kNN-DREMI scores in the Chondrocyte Differentiation ontology (GO:0002062) for the union of the *HACNS1* RL and *Gbx2* kNN-DREMI analysis gene lists are colored in red and labeled. Dotted lines indicate the top 20% of values for each dataset. **C** Heatmap showing expression of genes belonging to the ontology “Chondrocyte Differentiation” (GO:0002062) in all *HACNS1* homozygous mesenchymal cells (Louvain clusters 1–4). Hierarchical clustering was used to determine the order of cells (in columns) and genes (in rows). The bar at the top of the heatmap shows *Gbx2*-positive and *Gbx2*-negative cells in red and gray, respectively. **D** Expression of selected genes in *Gbx2*-positive (red) versus *Gbx2*-negative (gray) mesenchymal cells belonging to Louvain clusters 1 and 2, partitioned by proximal-distal axis markers as follows: Proximal cells (Prox) are *Meis*1+, *Hoxd13*−, *Hoxa11*−; distal cells (Dist) are *Hoxd13*+, *Hoxa11*− and *Meis1*−; and intermediate cells (Mid) are all remaining *Hoxa11* + cells. Cells were randomly down-sampled to enable comparison of equal numbers of *Gbx2*-positive and *Gbx2*-negative cells. Larger red and gray dots respectively denote mean expression of each indicated gene in each group in *Gbx2*-positive and *Gbx2*-negative cells. All gene expression values shown in C and D were imputed using ALRA and centered and scaled using z-scores (see “Methods”)^81^.

To identify genes whose expression is associated with *Gbx2*, we used k-Nearest-Neighbors Conditional-Density Resampled Estimate of Mutual Information (kNN-DREMI), which computes scores quantifying the strength of the relationship between two genes^53,54^. Using kNN-DREMI scores, we ranked each gene expressed in *HACNS1* homozygous limbs by the strength of its association with *Gbx2*. To determine if genes associated with *Gbx2* were enriched in particular functions, we then performed Gene Set Enrichment Analysis (GSEA) on this set of ranked genes. We found that *Gbx2* expression was associated with genes in several limb development-related ontologies, including “Cartilage Morphogenesis” (Kolmogorov–Smirnov (KS) *P* = 1.61 × 10^−3^) and “Regulation of Chondrocyte Differentiation” (KS P = 5.84 × 10^−3^); the latter overlapped considerably with “Collagen Fibril Organization” (KS *P* = 1.30 × 10^−4^) (Fig. 5A, Supplementary Data 5). These results indicate that in the *HACNS1* homozygous hindlimb, *Gbx2* is co-regulated with genes expressed in condensing mesenchymal cells destined to become chondrocytes (Fig. 5A).

We also used Manifold Enhancement of Latent Dimensions (MELD) to quantify the differences in the transcriptional profiles of *HACNS1* homozygous limb buds compared to the chimpanzee ortholog line and wild type. MELD is an unsupervised learning algorithm and is therefore an orthogonal approach that is naïve to our identification of *Gbx2* as the target of *HACNS1*^55^. MELD uses graph signal processing to quantify the relative likelihood of observing each cell in each of multiple experimental conditions based on its transcriptional profile. In this case, MELD is used to quantify the relative likelihood (RL) of observing a cell in the *HACNS1* hindlimb versus the chimpanzee ortholog or wild type hindlimb. Rather than explicitly classifying genes as differentially expressed in one condition versus another, the RL value can be used to identify trends in gene expression across cells that are associated with the *HACNS1* knock-in condition^55^.

To identify overall gene expression patterns characteristic of *HACNS1* homozygous hindlimb bud cells, we used kNN-DREMI to associate gene expression with the *HACNS1* knock-in condition (*HACNS1* RL) calculated by MELD. We then used the resulting gene rankings to identify enriched biological functions via GSEA, as described above for *Gbx2* expression. We found that genes associated with both *HACNS1* RL and *Gbx2* expression converged on related biological processes. Performing GSEA using genes ranked by mutual information with *HACNS1* RL revealed significant enrichment of the “Chondrocyte Differentiation” ontology (KS *P* = 7.00 × 10^−5^), along with four other categories also significantly enriched in the *Gbx2* expression analysis: “Hindlimb Morphogenesis” (KS *P* = 1.42 × 10^−3^), “Cartilage Development Involved in Endochondral Bone Morphogenesis” (KS *P* = 1.11 × 10^−3^), “Collagen Fibril Organization” (KS *P* = 1.40 × 10^−5^), and “Positive Regulation of Phosphatidylinositol 3-kinase Signaling” (KS *P* = 4.40 × 10^−5^), of which the last two are also implicated in chondrocyte differentiation (Fig. 5A, Supplementary Data 6)^56,57^. “Collagen Fibril Organization” is the most significantly enriched GO term for genes associated with *HACNS1* RL and is the second most enriched for genes associated with *Gbx2* expression. The top GO term for genes associated with *Gbx2* expression, “Roof of Mouth Development”, (KS *P* = 1.10 × 10^−5^), shares >20% of its genes with “Embryonic Hindlimb Morphogenesis” (KS *P* = 1.18 × 10^−3^)^58^. This illustrates that many genes involved in limb development are also implicated in craniofacial development, and likely accounts for why craniofacial development-related GO terms were also enriched in our analysis.

These findings led us to examine the expression patterns of chondrocyte differentiation-related genes in *HACNS1* homozygous mesenchymal cells belonging to Louvain clusters 1–4 (Fig. 5C). We clustered *HACNS1* homozygous mesenchymal cells by humanized RL and *Gbx2* expression and examined the expression of the “Chondrocyte Differentiation” ontology genes within *Gbx2*-positive cells (Fig. 5C). This clustering analysis revealed higher expression of positive regulators of chondrocyte differentiation (e.g., *Sox9*, *Col2a1*, *Bmp2*, and *Runx2)* specifically in *Gbx2*-positive versus *Gbx2*-negative humanized mesenchymal cells, supporting that *HACNS1-*driven upregulation of *Gbx2* occurs in chondrogenic cells (Fig. 5C)^57,59–61^. We also identified a subset of *Gbx2*-positive humanized cells that were also positive for *Bmp4*, which is expressed in the apoptotic interdigital domains^62^. These findings suggest that upregulation of *Gbx2* is associated with the interdependent pathways of digit condensation and interdigital cell fate specification required for digit morphogenesis.

The gain of a *Gbx2* anterior proximal hindlimb expression domain in *HACNS1* knock-in mice suggests that *Gbx2* upregulation may impact development of multiple segments in the limb (Fig. 3A, B). To explore this further, we characterized the distribution of *Gbx2* expression along the proximal-distal (PD) axis in the *HACNS1* homozygous hindlimb single-cell dataset. We defined three groups of mesenchymal cells along the (PD) axis based on expression of the patterning markers shown in Fig. 4: distal (*Hoxd13*+, *Hoxa11−, Meis1*−); proximal (*Hoxd13−, Hoxa11−, Meis1*+); and intermediate (all remaining *Hoxa11*+) cells. *Gbx2* was expressed in all three subtypes (Fig. 5D, top). We also examined expression of several “Chondrocyte Differentiation” ontology genes (selected from Fig. 5C) in *Gbx2* + and *Gbx2*- cells in each subtype. Cells expressing *Gbx2* also expressed chondrocyte differentiation markers along the PD axis, with a subset of markers showing modestly increased expression in *Gbx2* + cells (Fig. 5D, middle and bottom). Together, these results link *Gbx2* upregulation to chondrocyte differentiation in multiple developing regions of the hindlimb.

### Morphometric analysis of *HACNS1* knock-in mouse limbs

To determine if *Gbx2* upregulation and downstream transcriptional changes in *HACNS1* limb buds affect digit formation or overall limb morphology, we performed morphometric analysis of skeletal preparations for embryos homozygous for *HACNS1*, the chimpanzee ortholog, and wild type embryos (Methods). We performed morphometric analysis at E18.5 in order to capture any major phenotypic effects of targeted humanization that occurred by the end of embryonic skeletogenesis^22^. We did not detect gross morphological differences among genotypes; the three major limb segments (autopod, zeugopod, and stylopod) were present in both *HACNS1* skeletons and chimpanzee ortholog skeletons (Supplementary Fig. 6). We also examined digit length (normalized to body size based on the length of the ossified humerus), and intradigital (phalange to metacarpal or metatarsal length) and interdigital ratios. Again, we found no significant differences in digit length or autopod proportions between genotypes (Supplementary Fig. 6, Supplementary Data 7–9; Source Data). Although these analyses failed to detect morphological differences, it is possible that subtle phenotypes do indeed exist in the *HACNS* homozygous limb, which may be revealed in future studies.

## Discussion

Understanding how uniquely human genetic changes altered developmental processes is essential to understanding the evolution of our species. Here we investigated the role of the Human Accelerated Region *HACNS1* in human limb evolution by directly interrogating its biological functions in a genetically humanized mouse model. This in vivo approach enabled us to identify spatial and temporal changes in gene expression driven by *HACNS1* and to characterize the specific cell types affected by these changes, providing insight into the developmental processes modified due to human-specific alterations in enhancer activity.

First, we determined that *HACNS1* is active in the mouse genomic context, recapitulating the significant level of H3K27ac marking previously observed in the developing human limb in the *trans-*regulatory environment of the developing mouse limb. Second, we found that *Gbx2* exhibited increased promoter activity in the *HACNS1* homozygous limb bud, strongly supporting that it is regulated by *HACNS1*, and then demonstrated that *HACNS1* drives robust changes in *Gbx2* expression in the forelimb and hindlimb bud. These findings support the long-standing hypothesis that discrete regulatory changes altering expression of pleiotropic developmental regulators in specific tissues contribute to the evolution of phenotypic differences—in the case of this study, molecular phenotypes—across species^20,21^. Third, by performing scRNA-seq, we identified the spectrum of distinct cell types that show upregulation of *Gbx2* in the developing limb, as defined by transcriptome signatures. This analysis not only established that *Gbx2* is expressed in mesenchymal cells in the limb bud, but also placed these cells in the developmental process of chondrogenesis. By both characterizing *Gbx2*-positive cells in the *HACNS1* homozygous limb bud and identifying overall expression trends associated with the *HACNS1* knock-in condition without reference to *Gbx2* expression, we implicated changes in *Gbx2* regulation in chondrocyte differentiation, a critical process in skeletal development.

We found that the human-specific gain of function in *HACNS1* drives quantitative, spatial, and temporal changes in *Gbx2* expression in the *HACNS1* knock-in mouse limb bud. One hypothesis consistent with these findings is that *HACNS1* acts to both spatially expand and prolong *Gbx2* expression and its potential effects on chondrocyte differentiation in the developing limb. *Gbx2* is expressed in wild type mouse limb at E10.5, and the mouse ortholog of *HACNS1* is marked by H3K27ac at this time point, suggesting it is contributing to *Gbx2* regulation^27,63,64,65^. However, the mouse ortholog is annotated as a poised enhancer in the E11 limb bud, consistent with the weak expression of *Gbx2* we observe in E11-E12 wild type limb buds^65^. In contrast, *HACNS1* shows robust H3K27ac marking in the E11.5 limb, and *Gbx2* is strongly expressed in the E11 *HACNS1* limb bud. These results, coupled with our finding that *Gbx2* is primarily expressed in limb mesenchymal cells in all three genetic backgrounds we interrogated, suggests that the gain of function in *HACNS1* may be modifying an ancestral *Gbx2* regulatory program in the limb. The temporal extension of *Gbx2* expression we observed in *HACNS1* knock-in limb buds also raises the possibility that *HACNS1* contributes to human-specific heterochronic changes in this ancestral regulatory program. This is particularly intriguing given the protracted timing of human development compared to chimpanzee and mouse. Further insight into these questions will require deciphering the *Gbx2* regulatory network in both *HACNS1* knock-in and wild type limbs, including identifying downstream targets of GBX2 and upstream regulators of *HACNS1* and the orthologous mouse enhancer. We note that several of the transcription factors whose predicted binding sites are present only in *HACNS1* have been implicated in limb bud patterning and chondrogenesis at E11, including *Ets1* and *Gabpa*^66^, *Tfap2B*^67^, and *Runx2* (Supplementary Data 2)^68^. Additional longitudinal single-cell expression studies in the *HACNS1* knock-in limb may also reveal downstream transcriptional changes that could not be identified in the single time point we examine here.

The modest, but observable, differences in *Gbx2* expression in the chimpanzee ortholog line compared to wild type may be due to primate-rodent sequence changes that altered ancestral enhancer activity at the *HACNS1* locus, predating the human-specific changes we focused on in this study. Although *HACNS1* itself shows very high levels of sequence conservation with the chimpanzee and mouse orthologs, the flanking human and chimpanzee sequences we included in our knock-in models harbor multiple differences, including single nucleotide substitutions and gaps, relative to the endogenous mouse locus (Fig. 1B; Supplemental Note). These sequence changes may have contributed either to gain of function at the *HACNS1* locus during primate evolution, or to loss of function on the rodent lineage. Distinguishing between these mechanisms will require in vivo genetic studies of *HACNS1* orthologs from multiple primate, rodent, and outgroup species, and of primate and rodent ancestral orthologs inferred by ancestral sequence reconstruction.

Our morphological studies in the *HACNS1* mouse model also have several limitations that we note here. We did not identify major changes in skeletal morphology at E18.5 that were associated with targeted humanization, despite the large number of biological replicates we used in our analyses. However, this does not preclude subtle changes in limb length or other features in adult mice, or changes in soft tissues that would not be detected using skeletal preparations. Furthermore, we did not exhaustively characterize other tissues, including the pharyngeal arch and diencephalon, in which *Gbx2* has known developmental functions^26–29,31^. Although we did not observe overt craniofacial phenotypes in *HACNS1* knock-in mice, the dorsal expansion of *Gbx2* expression into the second pharyngeal arch that we detected in this background may result in more subtle developmental effects. The second arch contributes to the development of multiple structures, including the temporal styloid process, the hyoid bone, facial muscles, and the facial nerve^69,70^. Understanding which of these structures might be altered in *HACNS1* humanized mice could be explored in future studies.

We have shown that HAR knock-in mouse models represent a viable and fruitful approach for studying gene regulatory mechanisms relevant for human evolution within the complete genomic, tissue-level, and developmental framework of a living organism. We note that the lack of an overt morphological phenotype in the *HACNS1* model, despite the strong effects on gene expression that we observed, suggests it may be difficult to anticipate the full range of phenotypes HARs might generate in model systems based on their degree of acceleration. Nevertheless, it is not entirely surprising that that the *HACNS1-*driven molecular phenotypes we observed did not produce morphological phenotypes. Genetic changes in any one enhancer are unlikely to be sufficient to replicate human-specific morphological changes entirely in an experimental model. The evolution of uniquely human physical traits likely entailed modifications in the expression of many genes, potentially driven by multiple HARs and other human-specific genetic changes. Our study provides insight into how a single HAR alters gene regulation and expression at critical developmental time points, yielding an important entry point for understanding the larger developmental networks that changed during human limb evolution, of which *Gbx2* is a part. Knock-in mouse models offer the means to study additional HARs contributing to human-specific phenotypes at once, either through intercrossing mouse lines harboring edited unlinked loci or by iterative editing of one locus, allowing us to expand our understanding of human limb evolution. Our study thus establishes a framework for using genetically humanized mouse models to link sequence changes that arose on the human lineage to the unique traits that distinguish our species.

## Methods

### Mouse line generation and validation

All animal work was performed in accordance with approved Yale IACUC protocols (#2019–11167 and #2020–07271). The *HACNS1* and chimpanzee ortholog lines were generated at the Yale Genome Editing Center using standard gene targeting techniques in mouse ES cells^71^. C57BL/6J-*A*^*w−J*^/J mouse ES cells, generated by the Yale Genome Editing Center from C57BL/6J-*A*^*w−J*^/J mice obtained from The Jackson Laboratory (RRID:IMSR_JAX:000051), were edited by electroporation of a GFP cloning vector containing human (1241 bp) or chimpanzee (1240 bp) sequence flanked by C57BL/6 J mouse sequence homology arms, floxed pPGKneo vector, and diphtheria toxin sequence (Supplementary Fig. 1A)^72^. The genomic coordinates of the human (hg19; https://www.ncbi.nlm.nih.gov/assembly/GCF_000001405.13/), chimpanzee (panTro4; https://www.ncbi.nlm.nih.gov/assembly/GCF_000001515.6/), and mouse (mm9; https://www.ncbi.nlm.nih.gov/assembly/GCF_000001635.18/) sequences used in the editing constructs, including the mouse homology arm sequences, are listed in Supplementary Data 1^73^. Positive clones were karyotyped and only clones exhibiting a normal mouse karyotype were used for blastocyst injection. Resulting G0 chimeras were backcrossed to wild type C57BL/6 J (RRID: IMSR_JAX:000664) and crossed with an actin-Cre C57BL/6 J mouse line to remove the neo cassette. All mice used in our analysis were from F9 or later generations. Mice were maintained in a Yale Animal Resources Center (YARC) managed facility under a standard 12 h light/dark cycle and environmental monitoring according to YARC policies and procedures.

Genotyping primers specific to *HACNS1*, chimpanzee, and mouse orthologs are listed in Supplementary Data 10. Cloning primers listed in Supplementary Data 10 were used to amplify edited loci for cloning and Sanger sequencing for comparison to the hg19 or panTro4 sequence. Sanger sequencing data is available at http://noonan.ycga.yale.edu/noonan_public/Dutrow_HACNS1/. The sequence identity between the human (hg19, chr2:236773456-236774696) and chimpanzee alleles (panTro4, chr2B:241105291-241106530) is 98.2% (22 substitutions total, of which 15 are fixed in humans). Human-specific substitutions were defined as fixed if the derived allele frequency in dbSNP (v153) was >=0.9999 and if the ancestral sequence state was conserved between chimpanzee, rhesus macaque, orangutan, and marmoset. We provide a detailed analysis of sequence differences between the human, chimpanzee and mouse orthologs in the Supplemental Note (Supplementary Materials). *HACNS1-GBX2* locus TAD coordinates (hg19 chr2:236655261-237135261) are from H1 human ES cell Hi-C data; *HACNS1* and *GBX2* are present in the same TAD and *GBX2* is the only annotated protein-coding gene whose promoter is included in this TAD^32^.

Copy number verification qPCR was performed using genomic DNA from three F9 mice from each line using Power SYBR Green Mastermix (Thermo Fisher Scientific #4368577) and the StepOnePlus Real-Time PCR System (Applied Biosystems) with primers listed in Supplementary Data 10. All biological replicates of each genotype were run in triplicate and Ct values of each were normalized to a control region on a different chromosome (see Supplementary Data 10). Primary qPCR results are available as Source Data.

### Chromatin Immunoprecipitation, ChIP-qPCR and ChIP-seq

Tissue for chromatin preparation was collected from E11.5 forelimb and hindlimb bud pairs or pharyngeal arch tissue from *HACNS1* and chimpanzee ortholog line heterozygous crosses to obtain pooled, litter matched limb bud or pharyngeal arch samples for all three genotypes (*HACNS1* homozygous, chimpanzee ortholog line, and wild type). Two biological replicates were used per genotype per tissue, each with tissue pooled from three embryos. Pooled tissue was crosslinked and sonicated as previously described^74^. Chromatin for each genotype, tissue, and replicate was used for H3K27ac or H3K4me2 immunoprecipitation with 7.5 μg antibody and ~5 μg tissue per ChIP assay using Active Motif #39133 (RRID: AB_2561016) and Active Motif #39913 (RRID: AB_2614976) as previously described^74,75^. ChIP-qPCR was performed using Power SYBR Green Mastermix (Thermo Fisher Scientific #4368577) with primers listed in Supplementary Data 11. Samples were sequenced (2 × 100 bp) using standard Illumina protocols on an Illumina HiSeq 4000 (RRID: SCR_016386). To control for batch effects, all samples of the same tissue type were multiplexed and sequenced on a single lane.

Reference genomes edited to replace the mouse ortholog of *HACNS1* with the human or chimpanzee sequence were built using Bowtie (v2.2.8; RRID: SCR_005476)^76^. ChIP-seq raw reads were aligned to the mm9, mm9 with chimpanzee ortholog, or humanized mm9 reference genome using Bowtie with -sensitive and -no-unal settings. GC composition was assessed using fastQC and showed that GC content and bias were consistent across all experiments^77,78^. Tag directories for each experiment were generated using makeTagDirectory in HOMER with default settings and standard normalization to 10 million tags, and were used to generate bigwig files for visualization with makeUCSCfile^23^. All peaks were called with HOMER (v4.9.1 RRID: SCR_010881) using default settings for -histone (IP vs input fold change = 4, *p* = 0.0001, peak size = 500, minDist = 1000)^23^. All differential peaks were called with DESeq2 implemented in HOMER using getDifferentialPeaksReplicates.pl with default settings (fold change cutoff = 2, FDR cutoff = 5%); briefly, reads from each comparison are pooled, with ChIP and inputs pooled separately, such that new peaks are called and used for quantitative comparison between genotypes^23,24^. The complete datasets of all peaks tested in differential analyses can be found at http://noonan.ycga.yale.edu/noonan_public/Dutrow_HACNS1/.

### RNA extraction and RT-qPCR

E11-E12 embryos were collected from six *HACNS1* homozygous, chimpanzee ortholog line, or wild type litters generated by crossing homozygous animals for each line. All embryos within each genotype group were ordered based on stage (>70 total embryos) and were divided into six timepoint groups per genotype consisting of forelimb or hindlimb buds from 4-6 pooled embryos per time point per genotype per tissue. RNA was purified using the Qiagen miRNeasy Kit (#74106). Invitrogen Superscript III Reverse Transcription Kit (#18080-051) was used to prepare cDNA from each sample. qPCR with the resulting cDNA was performed using Power SYBR Green Mastermix (Thermo Fisher Scientific #4368577). All samples were analyzed in triplicate using primers listed in Supplementary Data 12 and Ct values of *Gbx2* were normalized to *Hprt1*. Primary RT-qPCR results are available as Source Data.

### Whole mount in situ hybridization

E11-E12 mouse embryos were collected from *HACNS1* homozygous (*n* = 7 litters), chimpanzee ortholog line (*n* = 8 litters), and wild type (*n* = 12 litters) homozygous crosses. Embryos were fixed and hybridized with the same preparation of antisense *Gbx2* mRNA probe under identical conditions as previously described^78,79^. The *Gbx2* probe used for hybridization contains the full mouse consensus CDS sequence (CCDS15150.1); NCBI CCDS Release 23; https://www.ncbi.nlm.nih.gov/projects/CCDS/CcdsBrowse.cgi?REQUEST=ALLFIELDS&DATA=CCDS15150.1&ORGANISM=10090&BUILDS=CURRENTBUILDS (NCBI CCDS Release 23 CCDS15150.1). The 178 embryos (55 from the *HACNS1* knock-in line, 52 from the chimpanzee ortholog line, and 71 from wild type) were divided into temporally-ordered sextiles within the E11-E12 window (~40–48 somites, although we did not rely on somite counts for staging) based on measurement of crown-rump length for each individual embryo^35^. For the data shown in Fig. 3B, embryos were assessed for staining pattern by three individuals blinded to genotype under a stereo microscope (Leica S6D). For the data shown in Supplementary Fig. 3A, B embryos were annotated by a single scorer blinded to genotype. The scoring scheme was based on previous studies, notably to assess whole-mount gene expression patterns as described in the VISTA Enhancer Browser (http://enhancer.lbl.gov/)^10,34,36^. Embryos were assigned to one of eleven categories of *Gbx2* expression pattern based on the anterior-posterior and proximal-distal localization of staining as well the intensity (strong versus weak) of staining: 1: anterior and posterior (AP); 2: anterior distal and posterior distal (APD); 3: distal (D); 4: anterior distal (AD); 5: anterior (A); 6: weak anterior and posterior (APL); 7: weak anterior (AL); 8: weak distal (DL); 9: weak anterior and posterior distal (APDL); 10: weak anterior distal (ADL); 11: no staining (N). Categories were merged for clarity in Fig. 3B in the following manner: categories 1–3: anterior and posterior; categories 4–5: anterior only; categories 6–10: weak staining. See Fig. 3B for representative images of staining patterns illustrating the scoring scheme used for qualitative assessment of expression. Representative images were taken using a Zeiss Stemi 2000-C stereomicroscope fitted with an AxioCam MRc5 digital camera and Zeiss AxioVision software. Images and associated annotations are available as Source Data.

### Single-cell RNA-sequencing

#### Sample preparation

Tissue for scRNA-seq was collected at E11.5 from two human ortholog line homozygous litters, two chimpanzee ortholog line homozygous litters, and two wild type litters. Embryos were staged as previously described in order to obtain samples from stage-matched T3 embryos from each genotype. Left hindlimb buds from three embryos per genotype per replicate were pooled. Following dissection, the tissue was immediately placed in CMFSG saline–glucose solution (1x Calcium–magnesium-free phosphate buffered saline from Thermo Fisher Scientific #21-040-CV with 0.1% glucose from Corning 45% Glucose #45001-116) on ice. Gibco TrypLE Express digestion solution was used for cellular dissociation (Thermo Fisher Scientific # 2605010). The dissociation reaction was stopped using 1xDMEM (ATCC 30–2002) with 10% heat-inactivated Fetal Bovine Serum (Sigma-Aldrich #F4135). The dissociated cells were filtered through a 40 μM strainer and harvested by centrifugation at 4 °C. Cells were washed and resuspended in 1x Calcium–magnesium-free phosphate buffered saline (Thermo Fisher Scientific #21-040-CV) with 0.04% BSA (Sigma-Aldrich #SRE0036). Cell number and viability were estimated on a Countess II Automated Cell Counter prior to library preparation of 10,000 cells (estimated cell recovery from 16,000 input cells) per sample using Chromium Single Cell 3ʹ GEM, Library & Gel Bead Kit v3 (10X Genomics PN-1000075). Libraries were sequenced (2 × 75 bp) on an Illumina HiSeq 4000 (RRID: SCR_016386). To control for batch effects, all samples were multiplexed across all lanes. Count matrices were produced from raw sequencing data using the Cell Ranger v3.0.2 package from 10X Genomics (RRID: SCR_017344).

#### Data filtering and preprocessing

Matrices from the 10x Cell Ranger platform were filtered and preprocessed using Seurat v3.0.1 (RRID: SCR_016341)^38^. Prior to the generation of Seurat objects, *Xist* gene counts were eliminated in order to avoid clustering by sex within mixed sample populations. Genes expressed in fewer than 5 cells per sample were removed. Cells with greater than 7.5% or 2% counts from mitochondrial genes or hemoglobin genes, respectively, were removed. Cells with total gene count (nGene) z-scores less than -1 (corresponding to ~700 or fewer detected genes) or greater than 4 (corresponding to ~6000 or greater detected genes) were removed, as were cells with total UMI count (nUMI) z-scores greater than 7 (corresponding to ~50,000 or greater detected UMIs; see Supplementary Fig. 5). One chimpanzee ortholog line replicate was removed during pre-processing due to high overall mitochondrial gene expression, indicative of low viability. Prior to data integration, expression values from each sample were normalized based on library size for pre-processing purposes only using the Seurat tool NormalizeData^38^. Louvain clustering as implemented in Seurat was performed for pre-processing purposes only using FindVariableFeatures, ScaleData, RunPCA, FindNeighbors, and FindClusters in order to remove endothelial cell clusters (*Cd34*-positive and *Pf4*-positive), clusters characterized by aberrant mitochondrial gene expression (low *mt-Co1*), and transcriptionally distinct clusters containing fewer than 30 cells per sample^38,42^. The numbers of cells remaining after pre-processing for each sample are listed in Supplementary Data 13.

#### Data normalization and integration

All subsequent normalization and integration steps after pre-processing were performed with raw counts for all cells retained after pre-processing (see Supplementary Data 13). Cell cycle scores were computed using CellCycleScoring in Seurat to regress out the difference between G2M and S phases, effectively preserving differences between cycling and non-cycling cells while reducing differences related to cell cycle amongst proliferating cells^38^. In addition to cell cycle scores, percent mitochondrial gene expression and nUMI values were regressed using SCTransform (SCT) in order to reduce the effects of sequencing depth and minor differences in mitochondrial DNA expression related to viability^38,80^. All SCT normalized datasets containing all genes from individual samples were integrated using SelectIntegrationFeatures, PrepSCTIntegration, FindIntegrationAnchors, and IntegrateData^38,80^.

Following integration, the combined dataset was randomly down-sampled to contain a maximum of 10,000 cells per genotype prior to embedding and clustering using SubsetData in Seurat^38^. PCA, UMAP, and Louvain clustering were implemented in Seurat using RunPCA, RunUMAP, FindNeighbors, and FindClusters^38,41^. Percentages of cells belonging to each Louvain cluster are shown in Supplementary Data 13. Normalized data from all samples combined were used for imputation using ALRA with default settings for the purposes of data visualization as shown in Fig. 4A–D, Supplementary Fig. 4A, B, and Fig. 5C, D^81^. Marker gene expression was compared between ALRA-imputed and unimputed data to establish that imputation did not substantially distort marker gene expression patterns in our dataset (Supplementary Fig. 4, Supplementary Data 13). Data normalization and integration, UMAP embedding, and Louvain clustering were performed prior to imputation. The threshold for identifying *Gbx2*-positive cells was set as an imputed *Gbx2* expression value greater than 0.1. This threshold was also used for identifying percentages of marker gene-positive cells in unimputed and imputed data as shown in Supplementary Data 13. All gene expression scaling and centering for visualization purposes was performed on normalized imputed or unimputed data using the Seurat ScaleData function with default parameters (scale.max = 10)^38^.

#### MELD, MAGIC, kNN-DREMI analyses

Cells belonging to mesenchymal cell clusters (clusters 1–4, see Fig. 4A, C) from all genotypes were used for MELD, MAGIC, kNN-DREMI, and Gene Set Enrichment Analysis (GSEA). Scaled data matrices from the Seurat object integrated assay were loaded using scprep for MELD, MAGIC, and kNN-DREMI (https://github.com/krishnaswamylab/scprep). MELD and MAGIC both denoise scRNA-seq data using graphs to model cellular state space. The same graph signal was used for both MELD and MAGIC as calculated by graphtools (1.5.2) with n_pca = 20, decay = 40, and knn = 10. MELD was run on one-hot vectors for each genotype independently using default parameters^55^. MAGIC was performed using the same graph signal as MELD^54^. We used the kNN-DREMI implementation provided in scprep and kNN-DREMI was run on MAGIC-imputed data^53^. kNN-DREMI analysis was used in order to identify genes with expression levels associated with either *Gbx2* expression in humanized hindlimb or cells with increased humanized RL as calculated using MELD. MAGIC was employed only for the purpose of generating denoised gene expression values for kNN-DREMI analysis of gene-gene relationships but was not used for data visualization, clustering, or sample-associated density estimation using MELD.

#### Gene set enrichment analysis

GSEA was performed using topGO v.2.34.0 (RRID: SCR_014798) on all expressed genes that were ranked by *Gbx2-*DREMI or humanized RL-DREMI score from the aforementioned humanized mesenchymal cell kNN-DREMI analysis^82^. Significant nodes were identified using a Kolmogorov–Smirnov test and the algorithm = “elim” argument. Ontologies listed in Supplementary Data 5 and 6 are the top 30 nodes with fewer than 100 annotated genes (to remove non-specific categories) and at least one gene in the top 20% of DREMI scores. Heatmap hierarchical clustering was performed using pheatmap v1.0.12 (RRID: SCR_016418)^83^.

### Skeletal staining

E18.5 skeletons from two litters from each of *HACNS1* homozygous, chimpanzee ortholog line, and wild type homozygous crosses (*n* = 48 embryos) were stained with Alcian Blue and Alizarin Red as previously described^71^. Skeletons were imaged under a stereo microscope (Leica S6D) and measured by a single scorer blinded to genotype using ImageJ 2.0.0. Bone and cartilage lengths of the forelimb and hindlimb pelvic girdle, stylopod, zeugopod, and autopod were measured blinded to genotype using ImageJ 2.0.0. Forelimb measurements include metacarpals 1–5 (cartilage), proximal phalanges 1–5 (cartilage), intermediate phalanges 2–5 (cartilage), distal phalanges 1–5 (cartilage), scapula (bone and cartilage), humerus (bone and cartilage), radius (bone and cartilage), and ulna (bone and cartilage). Hindlimb measurements include metatarsals 1–5 (cartilage), proximal phalanges 1–5 (cartilage), intermediate phalanges 2–5 (cartilage), distal phalanges 1–5 (cartilage), tibia (bone and cartilage), femur (bone and cartilage), pelvis (cartilage), ilium (bone), ischium (bone), pubis (bone), fibula (bone), calcaneum (cartilage), and talus (cartilage). Digit length was calculated as the sum of all metacarpal/metatarsal and phalanx segments. Raw measurements and digit length were normalized to the length of ossified humerus or femur for forelimb or hindlimb digits, respectively. Phalange to metacarpal ratio was calculated as the ratio of the sum of the phalange lengths of each digit to the corresponding metacarpal/metatarsal segment. Interdigital ratios were calculated using raw digit lengths. Raw measurements and images are available as Source Data.

### ANOVA analysis for gene expression and morphometry

ANOVA analysis was performed with the lme4 package in R (RRID: SCR_015654) using default parameters to dissect the effects of genotype on limb segment length (morphometric data)^84^. We calculated the effects of genotype, litter, sex, forelimb versus hindlimb, digit number, and right versus left (RL) on normalized digit length, phalange to metacarpal ratio and interdigital ratio (Length Ratio ~ Genotype * (1 | Genotype/Litter) * Sex * Limb * Digit * (1 | RL) * (1 | Litter/Embryo) * (1 | Sex/Embryo) * (1 | *Genotype/Embryo))*. Correction for multiple comparisons was performed using the Benjamini & Hochberg method^85^.

### Statistics and reproducibility

All ChIP-seq findings were validated using ChIP-qPCR of both the sequenced samples as well as additional biological replicates. Specificity of H3K27ac and H3K4me2 antibodies was validated by the authors using dot blot analysis. Additional validation measures including dot blot analysis and ChIP-qPCR were performed by Active Motif (https://www.activemotif.com/documents/tds/39133.pdf and https://www.activemotif.com/documents/tds/39913.pdf). RT-qPCR results shown in Supplementary Fig. 3C were validated with additional biological and technical replicates. All attempts at replication were successful. No statistical methods were used to predetermine sample size for ChIP-seq and RT-qPCR analyses and no data were excluded from these analyses. All samples prepared for ChIP-seq, RT-qPCR, ISH, scRNA-seq, or morphometric analysis as shown in the final figures were treated identically and processed in parallel. No statistical methods were used to predetermine optimal sample sizes for morphometric and ISH analyses. Instead, morphometric studies and ISH analyses were done using large sample sizes: limb samples from 48 embryos for morphometry and over 100 embryos obtained from multiple litters for each genotype for ISH analyses. For ISH and morphometric analyses, no data were excluded from the analyses; missing data values indicate samples that could not be evaluated/measured due to damage to the specimen. One scRNA-seq replicate from the chimpanzee ortholog line was excluded based on high overall mitochondrial gene expression, indicative of low sample quality based on preestablished filtering metrics. Qualitative analysis of ISH results were performed using a blinded approach by randomizing embryo identification numbers prior to annotation as described in in the Methods. Morphometric data was collected blinded to genotype by randomizing sample identification numbers. ChIP-seq, RT-qPCR, and scRNA-seq were performed without group allocation blinding as all biological and technical replicates were processed identically and in parallel and no qualitative analyses were required for these experiments. We did not consider the sex of embryonic samples as a variable in our studies.

### Reporting summary

Further information on research design is available in the Nature Research Reporting Summary linked to this article.

## Supplementary information

Supplementary Information

Description of Additional Supplementary Files

Supplementary Data 1-13

Reporting Summary

## Data Availability

The Gene Expression Omnibus accession number for the data reported in this paper is “GSE141471”. The SRA accession number for the raw ChIP-seq and scRNA-seq data is “SRP234725” and the BioProject accession number is “PRJNA593575”. All other relevant data supporting the key findings of this study are available within the article and its Supplementary Information files or from the corresponding author upon reasonable request. Source data are provided with this paper.

## Source data

Source Data
