# Supplementary material for: Modeling uniquely human gene regulatory function via targeted humanization of the mouse genome
Source: Nat Commun. 2022 Jan 13;13:304. doi: 10.1038/s41467-021-27899-w (PMC8758698; doi:10.1038/s41467-021-27899-w)
Supplement: Supplementary file 1 — Supplementary Information [file 41467_2021_27899_MOESM1_ESM.pdf]

Supplementary Materials for

Modeling uniquely human gene regulatory function via targeted humanization of the mouse genome

Emily V. Dutrow[1†], Deena Emera[1‡], Kristina Yim[1], Severin Uebbing[1], Acadia A. Kocher[1], Martina Krenzer[1§], Timothy Nottoli[2,3], Daniel B. Burkhardt[1#], Smita Krishnaswamy[1,4], Angeliki Louvi[5,6], James P. Noonan[1,6,7]*

Affiliations:
1. Department of Genetics, Yale School of Medicine, New Haven, CT, 06510, USA.
2. Department of Comparative Medicine, Yale School of Medicine, New Haven, CT, 06510, USA.
3. Yale Genome Editing Center, Yale School of Medicine, New Haven, CT, 06510, USA.
4. Department of Computer Science, Yale University, New Haven, CT, 06520, USA.
5. Department of Neurosurgery, Yale School of Medicine, New Haven, CT, 06510, USA.
6. Department of Neuroscience, Yale School of Medicine, New Haven, CT, 06510, USA.
7. Department of Ecology and Evolutionary Biology, Yale University, New Haven, CT, 06520, USA.

†Present address: Cancer Genetics and Comparative Genomics Branch, National Human Genome Research Institute, National Institutes of Health, Bethesda, MD 20892, USA.

‡Present address: Center for Reproductive Longevity and Equality, Buck Institute for Research on Aging, Novato, CA 94945, USA.

§Present address: Neuroscience Research Training Program, Department of Psychiatry, Yale School of Medicine, New Haven, CT, 06510, USA.

#Present address: Cellarity, Cambridge, MA 02139

*Correspondence to: james.noonan@yale.edu

This file includes:

Supplemental Note

Supplementary Figs. 1 to 6

Other Supplementary Materials for this manuscript include the following:

Supplementary Data 1. Genomic Sequence Coordinates of Editing Construct Template DNA

Supplementary Data 2. Predicted Transcription Factor Binding Site Changes in *HACNS1*

Supplementary Data 3. ChIP-seq Significant Differential Peaks

Supplementary Data 4. scRNA-seq *Gbx2* Expression Summary

Supplementary Data 5. *Gbx2* kNN-DREMI GSEA Results

Supplementary Data 6. *HACNS1* Relative Likelihood kNN-DREMI GSEA Results

Supplementary Data 7. Normalized Digit Length ANOVA

Supplementary Data 8. Phalange to Metacarpal/Metatarsal Ratio ANOVA

Supplementary Data 9. Interdigital Ratio ANOVA

Supplementary Data 10. Oligonucleotides for Genotyping, Cloning, and Copy Number Analysis

Supplementary Data 11. Oligonucleotides Used for ChIP-qPCR

Supplementary Data 12. Oligonucleotides Used for RT-qPCR

Supplementary Data 13. scRNA-seq Sample Summary

Supplemental Note

To obtain further insight into primate-rodent sequence differences at the *HACNS1* locus, we conducted independent global pairwise alignments of the mouse, human and chimpanzee alleles using the EMBOSS Needle tool (Supplementary Data 1)[1]. We first considered the entire ~1.2 kb human or chimpanzee sequence introduced to replace the orthologous endogenous locus in each line. The sequence identity between the human (hg19, chr2:236773456-236774696) and chimpanzee alleles (panTro4, chr2B:241105291-241106530) in the edited mouse lines is 98.2% (22 sequence differences total, of which 15 are fixed in humans). The sequence identity between the human allele and the mouse ortholog replaced in the edited mouse lines (mm9, chr1:91610327-91611486) is 68.6% with 14.5% gapped positions. The sequence identity between the chimpanzee allele and the mouse ortholog is 70.8% with 12.9% gapped positions. The chimpanzee and mouse sequences are identical at 18 of the 22 substituted positions in the human and chimpanzee alleles mentioned above.

HACNS1 itself, as defined in Prabhakar *et al.* 2006, is highly conserved among sarcopterygian vertebrates (i.e., lobe-finned fishes and tetrapods), including chimpanzee and mouse[2]. In pairwise alignments, *HACNS1* and its mouse ortholog are 88.6% identical, with 2.2% gapped positions (including potential single nucleotide variants and segregating indels, which we did not exclude in this analysis). The chimpanzee and mouse orthologs are 91.2% identical, with 2.2% gapped positions. Therefore, the majority of the sequence differences between human and mouse, and between chimpanzee and mouse, that we discuss above, fall outside of this core region. It is possible that primate- or rodent-specific sequence differences within the *HACNS1* core interval or in the flanking regions included in our targeting scheme may have contributed to potential primate-specific or rodent-specific changes in the ancestral enhancer function of the *HACNS1* locus.

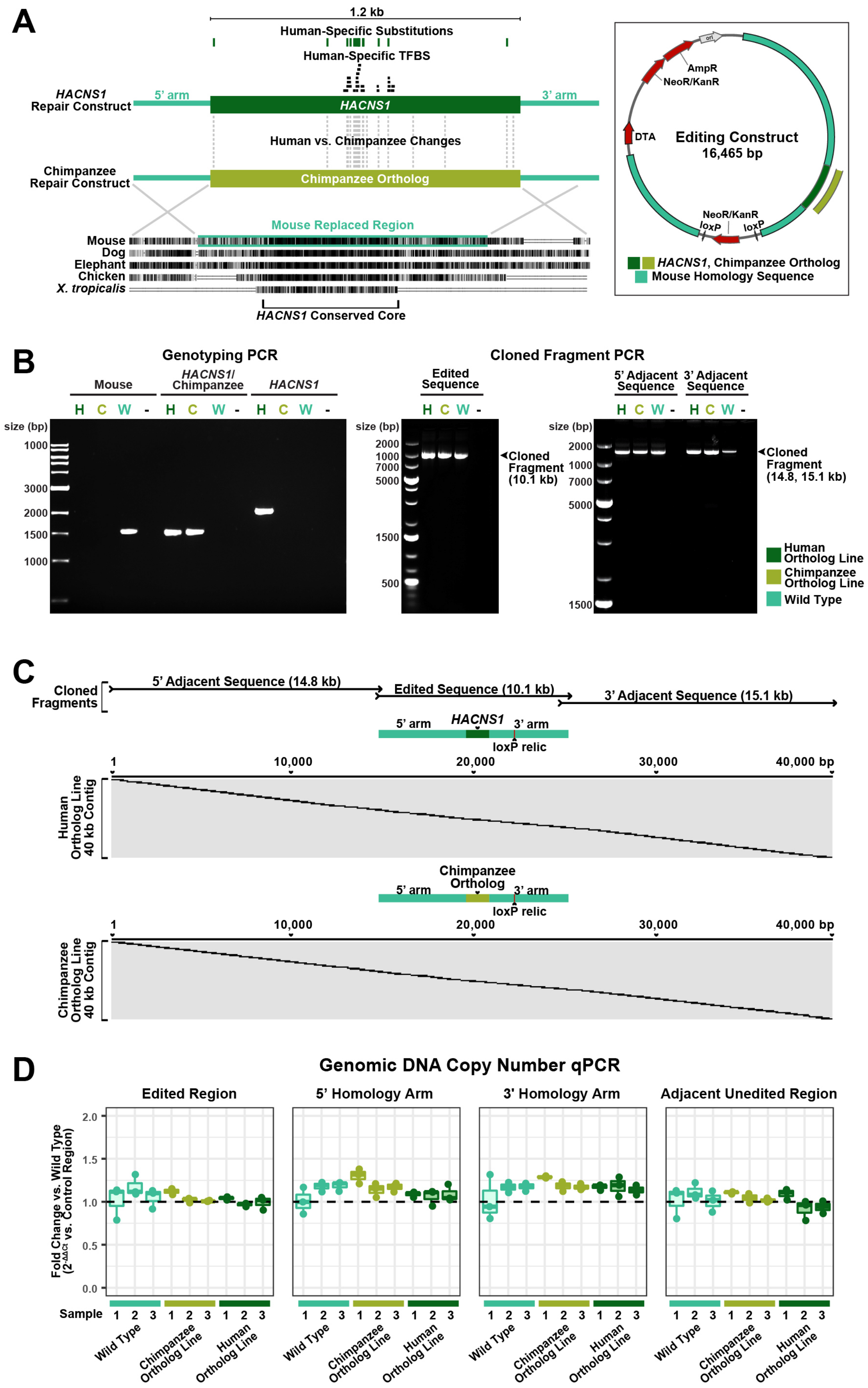
A
1.2 kb
Human-Specific Substitutions
Human-Specific TFBS
HACNS1 Repair Construct
5' arm
HACNS1
3' arm
Human vs. Chimpanzee Changes
Chimpanzee Repair Construct
Chimpanzee Ortholog
Mouse Replaced Region
Mouse
Dog
Elephant
Chicken
X. tropicalis
HACNS1 Conserved Core
AmpR
NeoR/KanR
DTA
Editing Construct 16,465 bp
NeoR/KanR
loxP
loxP
HACNS1, Chimpanzee Ortholog
Mouse Homology Sequence
B
Genotyping PCR
Mouse
HACNS1/ Chimpanzee
HACNS1
H C W -
size (bp)
Cloned Fragment PCR
Edited Sequence
5' Adjacent Sequence
3' Adjacent Sequence
Cloned Fragment (10.1 kb)
Cloned Fragment (14.8, 15.1 kb)
Human Ortholog Line
Chimpanzee Ortholog Line
Wild Type
C
Cloned Fragments
5' Adjacent Sequence (14.8 kb)
Edited Sequence (10.1 kb)
3' Adjacent Sequence (15.1 kb)
loxP relic
1
10,000
20,000
30,000
40,000 bp
Human Ortholog Line 40 kb Contig
Chimpanzee Ortholog
Chimpanzee Ortholog Line 40 kb Contig
D
Genomic DNA Copy Number qPCR
Edited Region
5' Homology Arm
3' Homology Arm
Adjacent Unedited Region
Fold Change vs. Wild Type (2^-ΔΔCt vs. Control Region)
Sample
Wild Type
Chimpanzee Ortholog Line
Human Ortholog Line

Supplementary Fig. 1. Development and validation of the *HACNS1* and chimpanzee ortholog mouse models. (A) *Left: HACNS1* and chimpanzee ortholog line editing constructs are shown with the orthologous replaced region in mouse genome aligned to other vertebrate species derived from the 100-way Multiz alignment in the UCSC hg19 assembly. Non-polymorphic, fixed human-specific substitutions are shown above the human construct and all human versus chimpanzee sequence differences are shown below. The transcription factor binding sites (TFBS) unique to *HACNS1* versus the chimpanzee ortholog shown are based on predictions of JASPAR core mammal motifs in the human and chimpanzee genomes (see also Supplementary Data 2)[3]. *Right:* Embryonic stem cell editing construct showing antibiotic resistance (NeoR/KanR), diphtheria toxin (DTA), mouse homology, and location of human or chimpanzee sequences. **(B)** *Left:* PCR products generated with primers specific to the mouse, both *HACNS1* and chimpanzee, and *HACNS1* only orthologs were used for genotyping of *HACNS1* homozygous (labeled as H), chimpanzee ortholog line (labeled as C), and wild type (labeled as W) mice from the F9 or later generation. *Middle:* PCR products were generated using primers outside the homology arms for Sanger sequencing of the edited locus. *Right:* PCR products were generated using primers anchored in the 5' homology arm and 14.8 kb upstream (5' adjacent sequence) and primers anchored in 3' homology arm and 15.1 kb downstream (3' adjacent sequence) for Sanger sequencing of the regions surrounding the editing locus. **(C)** Sanger sequencing contigs of cloned PCR products from (B), spanning the 40 kb region surrounding edited locus for the human ortholog line (*top*) and the chimpanzee ortholog line (*bottom*). **(D)** Human ortholog line, chimpanzee ortholog line, and wild type genomic DNA qPCR for primers specific to the edited region, 5' homology arm, 3' homology arm, and adjacent unedited region. All Ct values are normalized to a region on chromosome 5. Three biological replicate samples are shown per genotype and corresponding technical replicate data points (n = 3) overlay boxplots for each biological replicate denoting technical replicate quartiles and median. Primary results are available as associated Source Data.

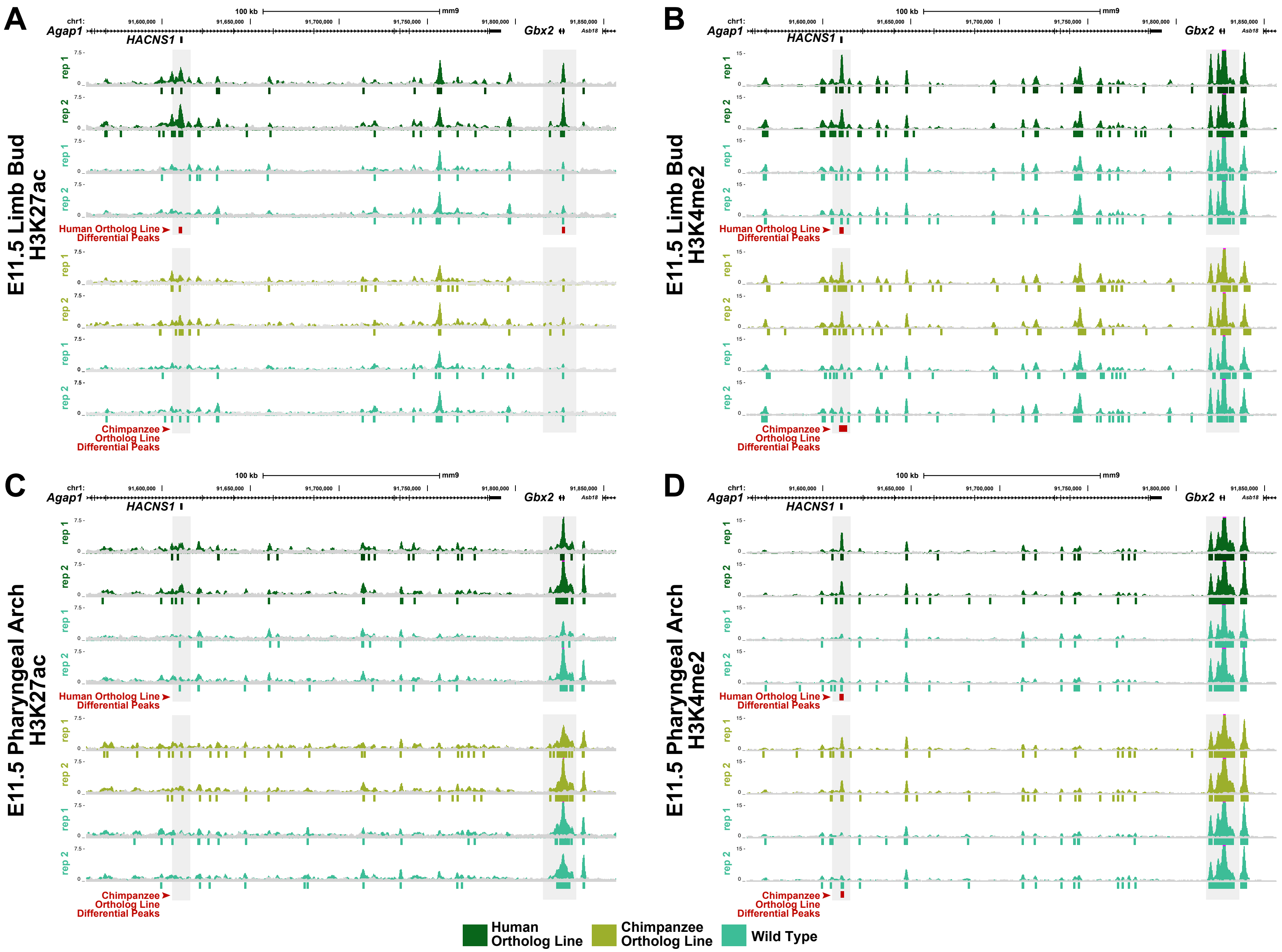

A
E11.5 Limb Bud H3K27ac
B
E11.5 Limb Bud H3K4me2
C
E11.5 Pharyngeal Arch H3K27ac
D
E11.5 Pharyngeal Arch H3K4me2
100 kb
mm9
chr1:
91,600,000
91,650,000
91,700,000
91,750,000
91,800,000
91,850,000
Agap1
Gbx2
Asb18
HACNS1
rep 1
rep 2
Human Ortholog Line Differential Peaks
Chimpanzee Ortholog Line Differential Peaks
Human Ortholog Line
Chimpanzee Ortholog Line
Wild Type

Supplementary Fig. 2. H3K37ac and H3K4me2 ChIP-seq analyses in limb bud and pharyngeal arch. Normalized H3K27ac (*left*) and H3K4me2 (*right*) epigenetic signals in the region spanning the full *HACNS1-Gbx2* locus for two biological replicates per genotype for E11.5 limb bud **(A, B)** and pharyngeal arch **(C, D)**. All corresponding input signal tracks are shown overlayed in gray. The location of the edited *HACNS1* locus relative to nearby genes is shown above each track group with a black bar directly below the corresponding UCSC mm9 genome track. *HACNS1* and *Gbx2* loci are highlighted in gray. All significant peaks are represented by genotype-specific colored bars below the signal tracks for the human ortholog line (in dark green), chimpanzee ortholog line (in olive), and litter-matched wild type (in teal). Peak calls showing significant signal increases between the human ortholog line and litter-matched wild type, or chimpanzee ortholog line and litter-matched wild type, are shown as red squares below each track group. Detailed differential peak information is available in Supplementary Data 3.

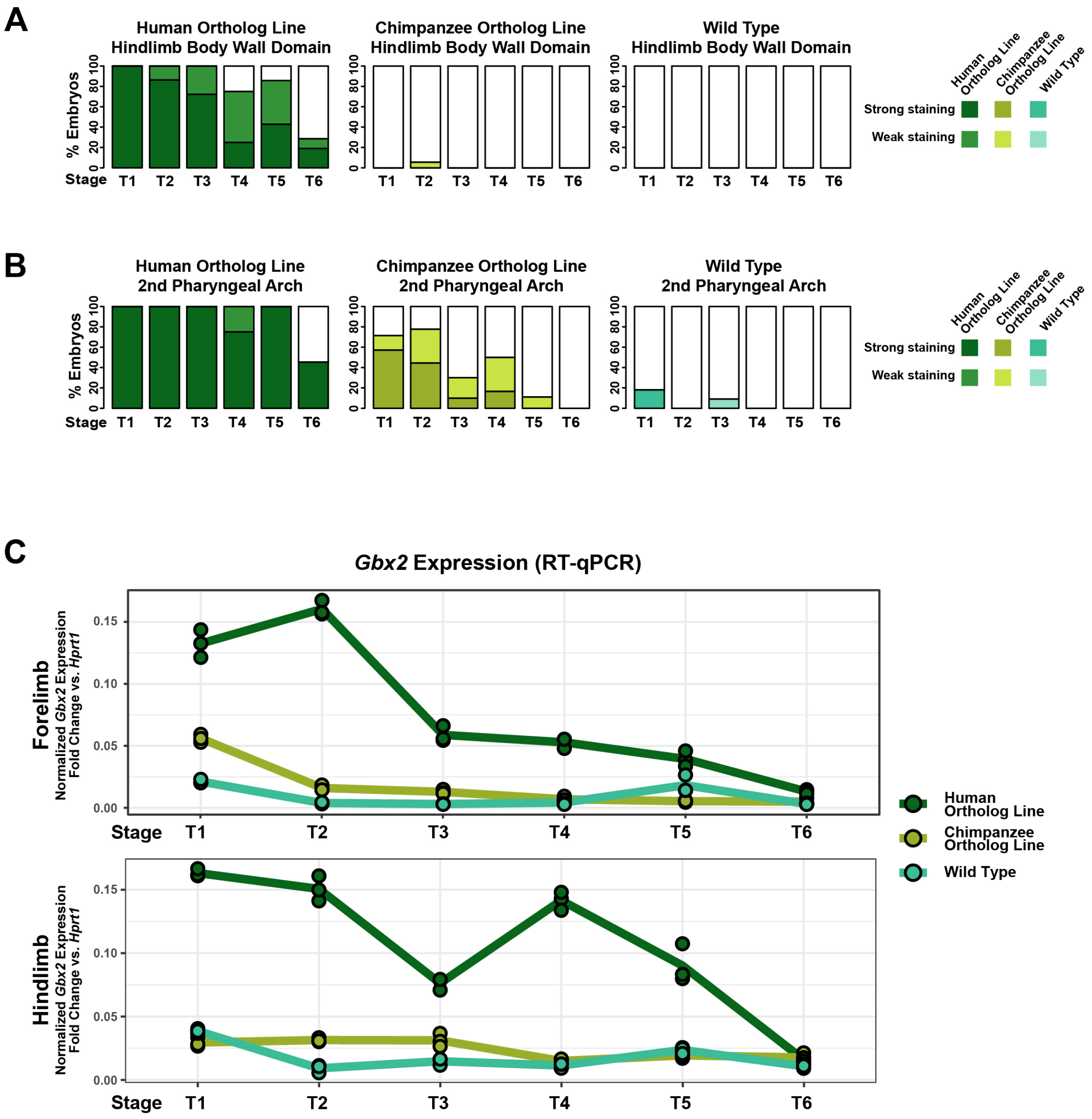
A
Human Ortholog Line
Hindlimb Body Wall Domain
Chimpanzee Ortholog Line
Hindlimb Body Wall Domain
Wild Type
Hindlimb Body Wall Domain
% Embryos
0 20 40 60 80 100
Stage T1 T2 T3 T4 T5 T6
Human Ortholog Line
Chimpanzee Ortholog Line
Wild Type
Strong staining
Weak staining
B
Human Ortholog Line
2nd Pharyngeal Arch
Chimpanzee Ortholog Line
2nd Pharyngeal Arch
Wild Type
2nd Pharyngeal Arch
% Embryos
Stage T1 T2 T3 T4 T5 T6
Strong staining
Weak staining
C
Gbx2 Expression (RT-qPCR)
Forelimb
Normalized Gbx2 Expression
Fold Change vs. Hprt1
0.00 0.05 0.10 0.15
Stage T1 T2 T3 T4 T5 T6
Hindlimb
Normalized Gbx2 Expression
Fold Change vs. Hprt1
Human Ortholog Line
Chimpanzee Ortholog Line
Wild Type

Supplementary Fig. 3. Additional analyses of *Gbx2* expression patterns, related to Figure 3. **(A)** Annotation results for presence or absence of hindlimb bud body wall domain in unique biological samples are shown for each genotype (n=103 human ortholog line; n=103 chimpanzee ortholog line; n=137 wild type). Strong versus weak staining is denoted by darker versus lighter shade, (See Fig. 3B for example image). **(B)** Annotation results for presence or absence of 2nd pharyngeal arch staining domain in unique biological samples are shown for each genotype (n=55 human ortholog line; n=52 chimpanzee ortholog line; n=71 wild type). Strong versus weak staining is denoted by darker versus lighter shade. Annotations in panels B and C were collected by a single scorer blinded to genotype and are available as associated Source Data. **(C)** Normalized *Gbx2* expression in pooled *HACNS1* homozygous*,* chimpanzee ortholog line, and wild type forelimb and hindlimb tissue across all six timepoints measured using RT-qPCR. Three technical replicates are plotted per genotype per timepoint with a line plotted denoting the mean value across technical replicates. Primary experimental results are available as associated Source Data.

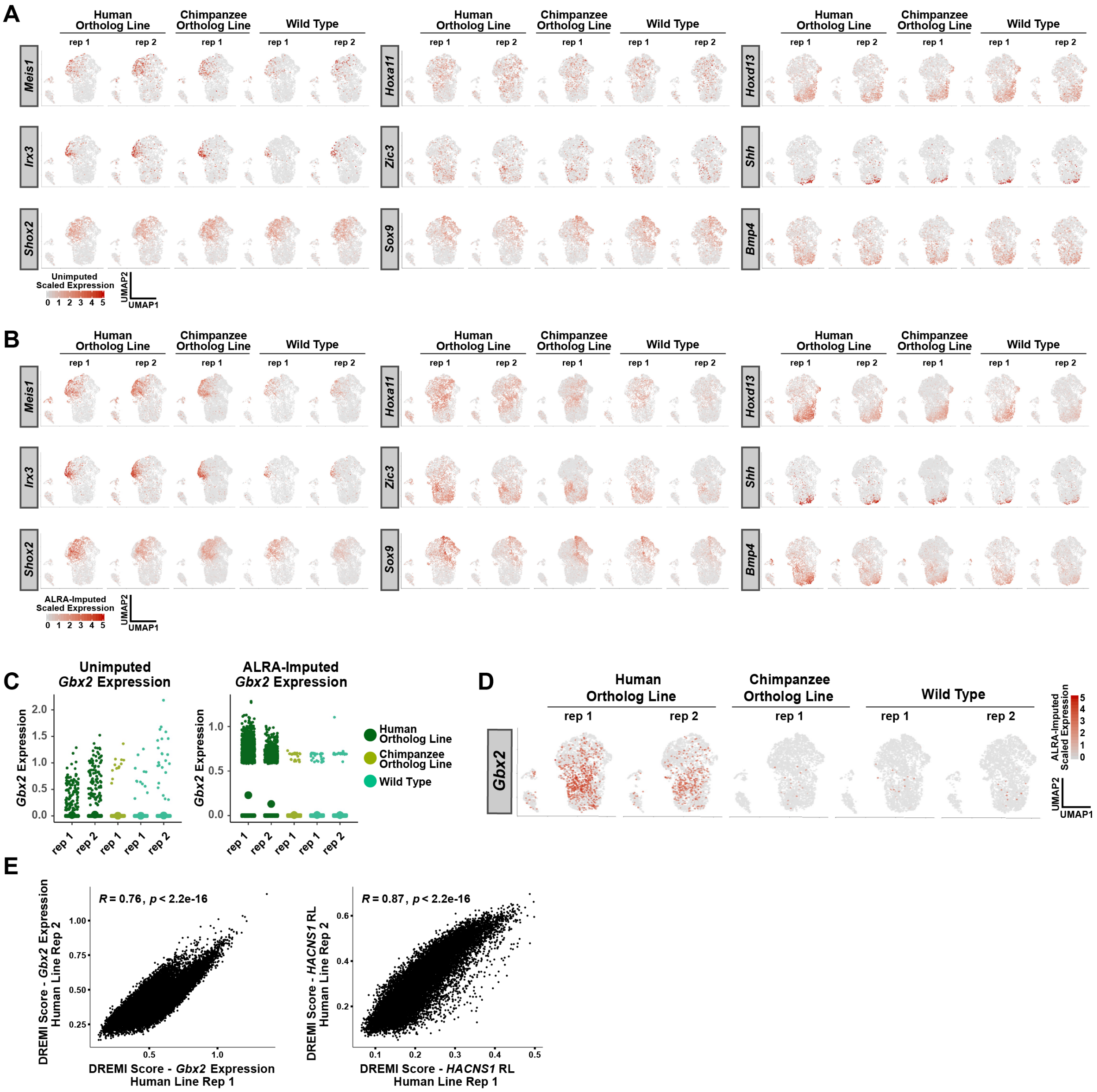
A
B
C
D
E
Human Ortholog Line
Chimpanzee Ortholog Line
Wild Type
rep 1
rep 2
Meis1
Irx3
Shox2
Hoxa11
Zic3
Sox9
Hoxd13
Shh
Bmp4
Unimputed Scaled Expression
ALRA-Imputed Scaled Expression
UMAP1
UMAP2
Unimputed Gbx2 Expression
ALRA-Imputed Gbx2 Expression
Gbx2 Expression
Gbx2
R = 0.76, p < 2.2e-16
R = 0.87, p < 2.2e-16
DREMI Score - Gbx2 Expression Human Line Rep 1
DREMI Score - Gbx2 Expression Human Line Rep 2
DREMI Score - HACNS1 RL Human Line Rep 1
DREMI Score - HACNS1 RL Human Line Rep 2

Supplementary Fig. 4. *Gbx2* and developmental marker expression in HACNS1 homozygous, chimpanzee ortholog line, and wild type E11.5 hindlimb bud replicates. (**A**) UMAP embedding of hindlimb bud cells from human ortholog line, chimpanzee ortholog line, and wild type replicates showing conserved expression of representative proximal-distal (*top row*), anterior-posterior (*middle row*), and chondrogenesis-apoptosis marker genes (*bottom row*). Gene expression data are unimputed and library size-normalized and were centered and scaled using z-scores for plotting (see Methods)[4]. See text and Fig. 4B for details on marker genes. **(B)** UMAP embedding of marker gene expression imputed using ALRA and centered and scaled using z-scores)[4]. See also Supplementary Data 13. **(C)** *Left*: Unimputed, library size-normalized *Gbx2* expression values by replicate. *Right*: ALRA-imputed *Gbx2* expression values by replicate. Dots indicate mean expression for each sample. **(D)** UMAP embedding of hindlimb bud cells from human ortholog line, chimpanzee ortholog line, and wild type replicates showing *Gbx2* expression values imputed using ALRA and centered and scaled using z-scores (see Methods)[4]. **(E)** DREMI scores for association with *Gbx2* (left) or relative likelihood of the *HACNS1* knock-in condition (*HACNS1* RL, right) for genes expressed in human ortholog line replicate 2 versus replicate 1. Spearman correlation values (two-sided) are shown for each plot with associated P values as calculated using the `cor` function in R (v3.5.0).

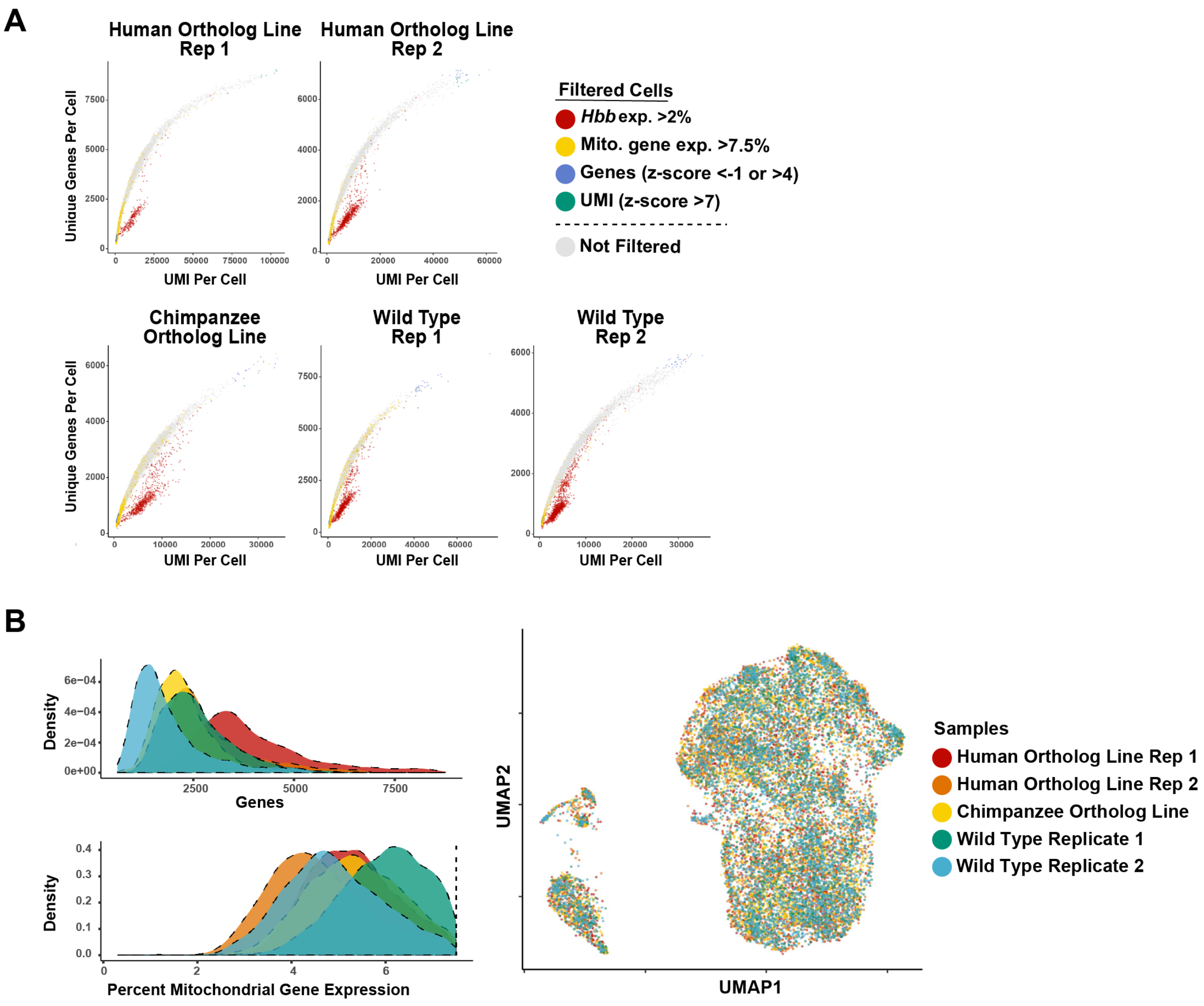
A
Human Ortholog Line Rep 1
Human Ortholog Line Rep 2
Unique Genes Per Cell
UMI Per Cell
Filtered Cells
Hbb exp. >2%
Mito. gene exp. >7.5%
Genes (z-score <-1 or >4)
UMI (z-score >7)
Not Filtered
Chimpanzee Ortholog Line
Wild Type Rep 1
Wild Type Rep 2
B
Density
Genes
Percent Mitochondrial Gene Expression
UMAP1
UMAP2
Samples
Human Ortholog Line Rep 1
Human Ortholog Line Rep 2
Chimpanzee Ortholog Line
Wild Type Replicate 1
Wild Type Replicate 2

Supplementary Fig. 5. Sample quality metrics in *HACNS1* homozygous, chimpanzee ortholog line, and wild type E11.5 hindlimb bud replicates. (A) UMI per cell versus number of unique genes per cell for human ortholog line, chimpanzee ortholog line, and wild type scRNA-seq replicates before filtering. Points representing cells are colored by the indicated filtering criteria. **(B)** *Left*: Density plots showing genes per cell (top) and percent mitochondrial gene expression per cell (bottom) for each replicate after filtering. Dashed line indicates 7.5% mitochondrial DNA expression cutoff implemented in filtering. *Right:* UMAP embedding showing each replicate colored separately.

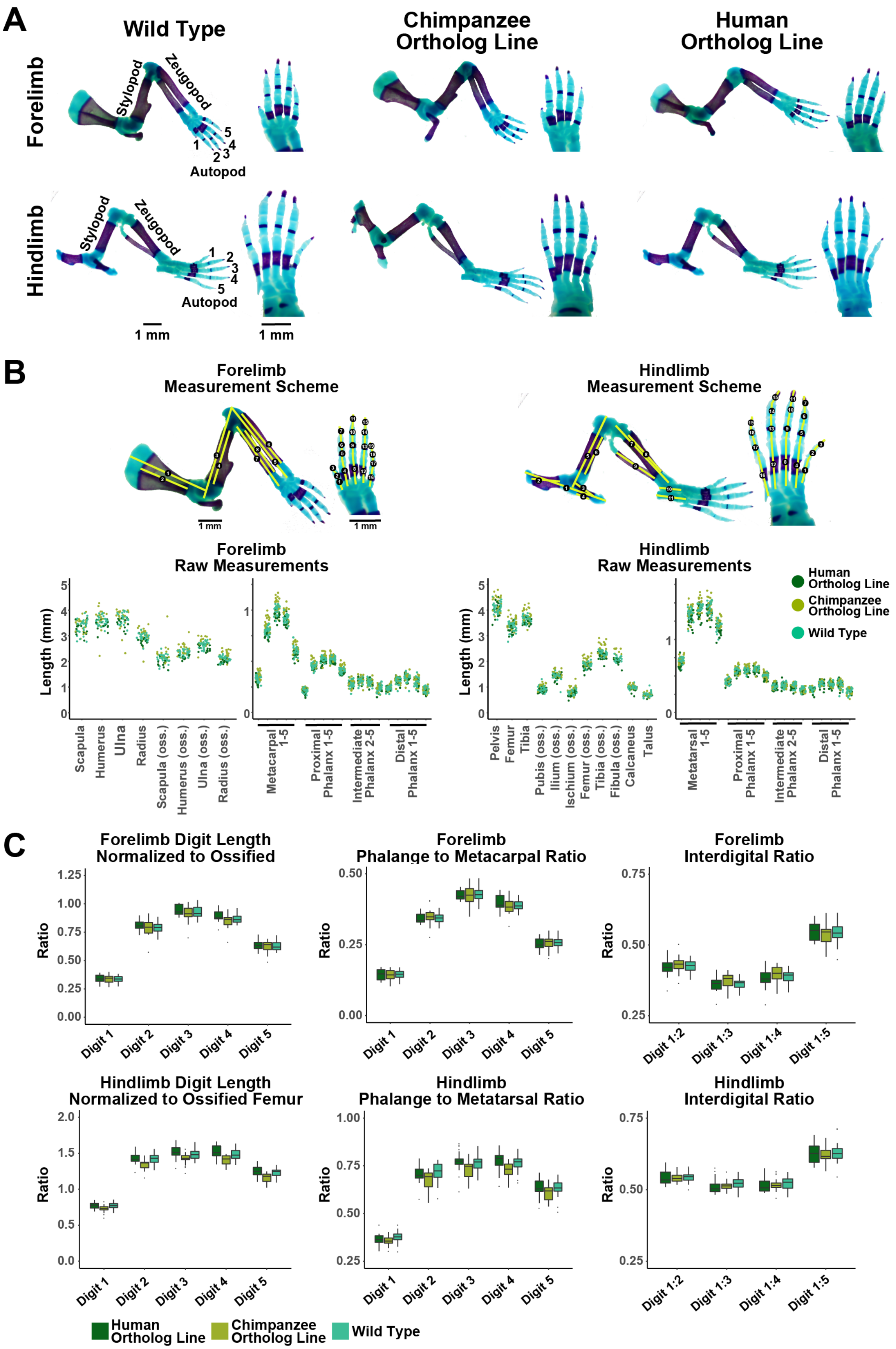
A
Wild Type
Chimpanzee Ortholog Line
Human Ortholog Line
Forelimb
Hindlimb
Stylopod
Zeugopod
Autopod
1 mm
B
Forelimb Measurement Scheme
Hindlimb Measurement Scheme
Forelimb Raw Measurements
Hindlimb Raw Measurements
Length (mm)
Human Ortholog Line
Chimpanzee Ortholog Line
Wild Type
Scapula
Humerus
Ulna
Radius
Scapula (oss.)
Humerus (oss.)
Ulna (oss.)
Radius (oss.)
Metacarpal 1-5
Proximal Phalanx 1-5
Intermediate Phalanx 2-5
Distal Phalanx 1-5
Pelvis
Femur
Tibia
Pubis (oss.)
Ilium (oss.)
Ischium (oss.)
Femur (oss.)
Tibia (oss.)
Fibula (oss.)
Calcaneus
Talus
Metatarsal 1-5
C
Forelimb Digit Length Normalized to Ossified
Forelimb Phalange to Metacarpal Ratio
Forelimb Interdigital Ratio
Hindlimb Digit Length Normalized to Ossified Femur
Hindlimb Phalange to Metatarsal Ratio
Hindlimb Interdigital Ratio
Ratio
Digit 1
Digit 2
Digit 3
Digit 4
Digit 5
Digit 1:2
Digit 1:3
Digit 1:4
Digit 1:5

Supplementary Fig. 6. Morphometric analyses of *HACNS1* homozygous, chimpanzee ortholog line, and wild type skeletons. (A) Representative images of E18.5 forelimbs and hindlimbs of the indicated genotypes stained with Alizarin Red (violet; bone) and Alcian Blue (blue; cartilage). Forelimb and hindlimb autopod, zeugopod, and stylopod are shown on the left, and numbers indicate digit identity. High magnification images of forelimb and hindlimb autopods are shown on the right. **(B)** *Top:* Measurement scheme for E18.5 skeleton morphometric analysis is shown with yellow lines denoting measured segments for forelimb zeugopod and stylopod cartilage and/or bone: scapula (1,2), humerus (3,4), ulna (5,6), radius (7,8); hindlimb zeugopod and stylopod cartilage and/or bone: pelvis (1), ilium (2), pubis (3), ischium (4), femur (5,6), tibia (7,8), fibula (9), talus (10), calcaneus (11), and metacarpal/metatarsal and phalange autopod segments (digit 1: 1-3; digit 2: 4-7; digit 3: 8-11; digit 4: 12-15; digit 5:16-19). *Bottom:* Raw data for all measured segments are plotted and colored by genotype. **(C)** Normalized digit length, phalange to metacarpal/metatarsal ratio, and interdigital ratio for forelimb and hindlimb digits are shown by genotype. Digit length is calculated as sum of all metacarpal/metatarsal and phalange segments. Forelimb and hindlimb digit lengths are normalized to ossified humerus and femur length of the same sample digit length, respectively. Boxplots denote quartiles and median for measurements of independent biological samples from the human ortholog line (n=23), chimpanzee ortholog line (n=25) and wild type (n=27). All measurements were collected by a single scorer blinded to genotype and are available as Source Data. For ANOVA analysis of morphometric data see Supplementary Data 7-9.

Supplemental References.

1. Madeira, F. *et al*. The EMBL-EBI search and sequence analysis tools APIs in 2019. *Nucleic Acids Res.* 47, W636–w641 (2019).
2. Prabhakar, S., Noonan, J. P., Pääbo, S. & Rubin, E. M. Accelerated evolution of conserved noncoding sequences in humans. *Science* 314, 786 (2006).
3. Uebbing, S. *et al*. Massively parallel discovery of human-specific substitutions that alter enhancer activity. *Proc. Natl. Acad. Sci. USA* 118, e2007049118 (2021).
4. Linderman, G. C., Zhao, J. & Kluger, Y. Zero-preserving imputation of scRNA-seq data using low-rank approximation. *bioRxiv*, 397588, https://doi.org/10.1101/397588 (2018).
