# Supplementary material for: Modeling uniquely human gene regulatory function via targeted humanization of the mouse genome
Source: Nat Commun. 2022 Jan 13;13:304. doi: 10.1038/s41467-021-27899-w (PMC8758698; doi:10.1038/s41467-021-27899-w)
Supplement: Supplementary file 2 — Description of Additional Supplementary Files [file 41467_2021_27899_MOESM2_ESM.docx]

Description of Additional Supplementary Files

Title: Supplementary Data 1

Description: Genomic Sequence Coordinates of Editing Construct Template DNA

Title: Supplementary Data 2

Description: Predicted Transcription Factor Binding Site Changes in HACNS1

Title: Supplementary Data 3

Description: ChIP-seq Significant Differential Peaks

Title: Supplementary Data 4

Description: scRNA-seq Gbx2 Expression Summary

Title: Supplementary Data 5

Description: Gbx2 kNN-DREMI GSEA Results

Title: Supplementary Data 6

Description: HACNS1 Relative Likelihood kNN-DREMI GSEA Results

Title: Supplementary Data 7

Description: Normalized Digit Length ANOVA

Title: Supplementary Data 8

Description: Phalange to Metacarpal/Metatarsal Ratio ANOVA

Title: Supplementary Data 9

Description: Interdigital Ratio ANOVA

Title: Supplementary Data 10

Description: Oligonucleotides for Genotyping, Cloning, and Copy Number Analysis

Title: Supplementary Data 11

Description: Oligonucleotides Used for ChIP-qPCR

Title: Supplementary Data 12

Description: Oligonucleotides Used for RT-qPCR

Title: Supplementary Data 13

Description: scRNA-seq Sample Summary
