# Supplementary material for: Modeling uniquely human gene regulatory function via targeted humanization of the mouse genome
Source: Nat Commun. 2022 Jan 13;13:304. doi: 10.1038/s41467-021-27899-w (PMC8758698; doi:10.1038/s41467-021-27899-w)
Supplement: Supplementary file 4 — Reporting Summary [file 41467_2021_27899_MOESM4_ESM.pdf]

nature research

Corresponding author(s): James P. Noonan

Last updated by author(s): December 8, 2021

Reporting Summary

Nature Research wishes to improve the reproducibility of the work that we publish. This form provides structure for consistency and transparency in reporting. For further information on Nature Research policies, see our Editorial Policies and the Editorial Policy Checklist.

Statistics

For all statistical analyses, confirm that the following items are present in the figure legend, table legend, main text, or Methods section.

n/a	Confirmed	
☐	☒	The exact sample size (*n*) for each experimental group/condition, given as a discrete number and unit of measurement
☐	☒	A statement on whether measurements were taken from distinct samples or whether the same sample was measured repeatedly
☐	☒	The statistical test(s) used AND whether they are one- or two-sided<br>*Only common tests should be described solely by name; describe more complex techniques in the Methods section.*
☐	☒	A description of all covariates tested
☐	☒	A description of any assumptions or corrections, such as tests of normality and adjustment for multiple comparisons
☐	☒	A full description of the statistical parameters including central tendency (e.g. means) or other basic estimates (e.g. regression coefficient) AND variation (e.g. standard deviation) or associated estimates of uncertainty (e.g. confidence intervals)
☐	☒	For null hypothesis testing, the test statistic (e.g. *F*, *t*, *r*) with confidence intervals, effect sizes, degrees of freedom and *P* value noted<br>*Give P values as exact values whenever suitable.*
☒	☐	For Bayesian analysis, information on the choice of priors and Markov chain Monte Carlo settings
☒	☐	For hierarchical and complex designs, identification of the appropriate level for tests and full reporting of outcomes
☒	☐	Estimates of effect sizes (e.g. Cohen's *d*, Pearson's *r*), indicating how they were calculated

Our web collection on statistics for biologists contains articles on many of the points above.

Software and code

Policy information about availability of computer code

Data collection	ImageJ 2.0.0; AxioVision software (Zeiss)
Data analysis	fastQC v0.11.5; Bowtie2 v2.2.8; HOMER v4.9.1; topGO v2.34.0; Cell Ranger v3.0.2; Seurat v3.0.1; scprep v0.10.0; MELD v0.2.3; MAGIC v1.5.0; graphtools 1.5.2; R Stats v3.5.0

For manuscripts utilizing custom algorithms or software that are central to the research but not yet described in published literature, software must be made available to editors and reviewers. We strongly encourage code deposition in a community repository (e.g. GitHub). See the Nature Research guidelines for submitting code & software for further information.

Data

Policy information about availability of data

All manuscripts must include a data availability statement. This statement should provide the following information, where applicable:

- Accession codes, unique identifiers, or web links for publicly available datasets
- A list of figures that have associated raw data
- A description of any restrictions on data availability

The Gene Expression Omnibus accession number for the ChIP-seq bigwig files, ChIP-seq peak files, and scRNA-seq count matrices is GSE141471 (https://www.ncbi.nlm.nih.gov/geo/query/acc.cgi?acc=GSE141471). The SRA accession number for the raw ChIP-seq and scRNA-seq data is SRP234725 (BioProject PRJNA593575). ChIP-seq data is associated with Fig. 2, S2. scRNA-seq data is associated with Fig. 4, 5, S4, S5. NCBI assembly and annotation data used in this paper are available at https://www.ncbi.nlm.nih.gov/assembly/GCF_000001635.18/ (MGSCv37/mm9), https://www.ncbi.nlm.nih.gov/assembly/GCF_000001405.13/ (GRCh37/hg19), https://www.ncbi.nlm.nih.gov/assembly/GCF_000001515.6/ (Pan_troglodytes-2.1.4/panTro4), and https://www.ncbi.nlm.nih.gov/projects/CCDS/CcdsBrowse.cgi?REQUEST=ALLFIELDS&DATA=CCDS15150.1&ORGANISM=10090&BUILDS=CURRENTBUILDS (NCBI CCDS Release 23 CCDS15150.1). In situ hybridization raw image files and associated data (Fig. 3, S3), and skeletal staining images and data (Fig. S6) have been provided in a Source Data file. Additional raw

data including ChIP differential peak analysis data (Fig. 2, S2) and Sanger sequencing data (Fig. S1) can be found at http://noonan.ycga.yale.edu/. The Vista Enhancer Browser is publicly available at http://enhancer.lbl.gov/. Access to all additional data needed to evaluate the conclusions in the paper is provided in the manuscript and the Supplementary Materials.

Field-specific reporting

Please select the one below that is the best fit for your research. If you are not sure, read the appropriate sections before making your selection.

- [x] Life sciences
- [ ] Behavioural & social sciences
- [ ] Ecological, evolutionary & environmental sciences

For a reference copy of the document with all sections, see nature.com/documents/nr-reporting-summary-flat.pdf

Life sciences study design

All studies must disclose on these points even when the disclosure is negative.

Sample size	No statistical methods were used to predetermine sample size for ChIP-seq, scRNA-seq, or RT-qPCR analyses. In order to minimize noise and provide consistent results with fewer samples, all biological replicates for scRNA-seq and ChIP-seq experiments were derived from pooled tissue from three embryos each (requiring litter-matching with wild type embryos for ChIP-seq). Similarly, the RT-qPCR experiment involved pooling tissue (4-6 embryos per genotype per tissue per time point) in order to minimize noise and provide more consistent results with fewer samples. This strategy enabled the simultaneous processing of over 70 embryos from 6 litters. Morphometric studies and ISH analyses were done using large sample sizes: limb samples from 48 embryos for morphometry and over 100 embryos obtained from multiple litters for each genotype for ISH analyses. ChIP-seq findings were supported by orthogonal methods as described below.
Data exclusions	One scRNA-seq replicate from the chimpanzee ortholog line was excluded based on high overall mitochondrial gene expression indicative of low viability based on pre-established filtering metrics. For ISH and morphometric analyses, no data were excluded from the analyses; missing data values indicate samples that could not be evaluated/measured due to damage to tissue.
Replication	In order to ensure reproducibility of the experimental findings, all experiments were performed in parallel and with identical treatment of biological samples. All ChIP-seq findings were validated using qPCR of both the sequenced samples as well as additional biological replicates. RT-qPCR results shown in Fig. S3 were validated with additional biological and technical replicates. All samples prepared for ChIP-seq, RT-qPCR, ISH, and scRNA-seq data as shown in the final figures were treated identically and in parallel. All attempts at replication were successful.
Randomization	The biological replicates for the ChIP and scRNA experiments all required pooling of tissue from multiple embryos. In order to assign tissues to biological replicates, all samples from individual embryos were randomly assigned identification numbers that allowed for random allocation into pooling groups.
Blinding	Qualitative analysis of ISH results were performed using a blinded approach by randomizing embryo identification numbers prior to annotation. Morphometric data was collected blinded to genotype using randomized identification numbers. ChIP-seq, RT-qPCR, and scRNA-seq were performed without group allocation blinding as all biological and technical replicates were processed identically and in parallel and no qualitative analyses were required for these experiments.

Reporting for specific materials, systems and methods

We require information from authors about some types of materials, experimental systems and methods used in many studies. Here, indicate whether each material, system or method listed is relevant to your study. If you are not sure if a list item applies to your research, read the appropriate section before selecting a response.

Materials & experimental systems

n/a	Involved in the study	
☐	☒	Antibodies
☐	☒	Eukaryotic cell lines
☒	☐	Palaeontology and archaeology
☐	☒	Animals and other organisms
☒	☐	Human research participants
☒	☐	Clinical data
☒	☐	Dual use research of concern

Methods

n/a	Involved in the study	
☐	☒	ChIP-seq
☒	☐	Flow cytometry
☒	☐	MRI-based neuroimaging

Antibodies

Antibodies used	H3K27ac and H3K4me2 immunoprecipitation were performed with 7.5 µg antibody and approximately 5µg tissue per ChIP assay using Active Motif #39133 (RRID: AB_2561016) and Active Motif #39913 (RRID: AB_2614976).
Validation	Specificity of H3K27ac and H3K4me2 antibodies was validated by the authors using dot blot analysis. Additional validation measures including dot blot analysis and ChIP-qPCR were performed by Active Motif (https://www.activemotif.com/documents/tds/39133.pdf ; https://www.activemotif.com/documents/tds/39913.pdf)

Eukaryotic cell lines

Policy information about cell lines

Cell line source(s)	C57BL/6J-Aw-J/J mouse ES cells were used for mouse line generation at the Yale Genome Editing Center.
Authentication	Positive clones were karyotyped and only clones of verified karyotype were microinjected. Cells produced agouti coat color in the resulting founders as expected.
Mycoplasma contamination	Cells were confirmed free of mycoplasma contamination.
Commonly misidentified lines (See ICLAC register)	NA

Animals and other organisms

Policy information about studies involving animals; ARRIVE guidelines recommended for reporting animal research

Laboratory animals	All animal work was performed in accordance with approved Yale IACUC protocols (#2019-11167 and #2020-07271). . Mice were maintained in a Yale Animal Resources Center (YARC) managed facility under a standard 12h light/dark cycle and environmental monitoring according to YARC policies and procedures. C57BL/6J mice were obtained from Jackson Laboratory (Stock No. 000664) for generation of edited lines and subsequent backcrossing. Pooled tissue from both male and female embryos was used in experiments. Males and females for timed matings and line propagation ranged in age from 2 months to 2 years.
Wild animals	No wild animals were used in this study.
Field-collected samples	No field collected samples were used in this study.
Ethics oversight	All animal work was performed in accordance with approved Yale IACUC protocols.

Note that full information on the approval of the study protocol must also be provided in the manuscript.

ChIP-seq

Data deposition

☒ Confirm that both raw and final processed data have been deposited in a public database such as GEO.

☒ Confirm that you have deposited or provided access to graph files (e.g. BED files) for the called peaks.

Data access links *May remain private before publication.*	GEO accession number: GSE141471; SRA accession number: SRP234725 (BioProject PRJNA593575). We released these data with our preprint so they are public.
Files in database submission	GEO submission contains bigwig files and peak files; SRA contains raw ChIP-seq and scRNA-seq data; ChIP differential peak analysis data can be found at http://noonan.ycga.yale.edu/noonan_public/Dutrow_HACNS1/ChIP_Differential_Analysis/
Genome browser session (e.g. UCSC)	N/A

Methodology

Replicates	Two biological replicates for each tissue and genotype for the humanized and chimpanzee ortholog line samples were used for sequencing. Four biological replicates were used from wild type in order to match litters for each humanized to wild type and chimpanzee ortholog line to wild type comparison. Each biological replicate contains tissue pooled from 3 embryos each. Differential peak analysis was performed using both biological replicates of each sample as implemented using getDifferentialPeaksReplicates.pl (HOMER v4.9.1). Results were validated using ChIP-qPCR for all biological replicates with three technical replicates.
Sequencing depth	Paired end reads (2x100bp) were generated for each sample. Samples were split across lanes by antibody only (H3K27ac, H3K4me2, and input) to avoid batch effects. We aimed for 40M read pairs per sample. Processed read statistics are available in individual peak file headers for each sample available at GEO accession GSE141471. Raw read counts are available at the linked SRA accession SRP234725.
Antibodies	H3K27ac and H3K4me2 immunoprecipitation were performed with 7.5 µg antibody and approximately 5µg tissue per ChIP assay using Active Motif #39133 (RRID: AB_2561016) and Active Motif #39913 (RRID: AB_2614976).
Peak calling parameters	Reads were aligned using bowtie2 (v2.2.8) with --sensitive and --no-unal and index files from mm9 or a custom mm9 index that includes the edited HACNS1 locus sequence (human or chimpanzee ortholog). Peaks were called using HOMER v.4.9.1 with findPeaks and the parameter -style histone. Replicating differential peaks were identified using HOMER v.4.9.1 getDifferentialPeaksReplicates.pl with parameters -DESeq2 -style histone.
Data quality	fastQC v0.11.5 was used to assess sequence quality. The average fraction of reads in peaks for H3K27ac and H3K4me2 samples were 0.35 and 0.61, respectively. Additional data quality metrics are available in individual peak file headers for each sample available at GEO accession GSE141471.

Software	bowtie2 v2.2.8; HOMER v.4.9.1; fastQC v0.11.5
